# Nuclei as mechanical bumpers during epithelial remodeling

**DOI:** 10.1083/jcb.202405078

**Published:** 2024-09-26

**Authors:** Noah F. de Leeuw, Rashmi Budhathoki, Liam J. Russell, Dinah Loerke, J. Todd Blankenship

**Affiliations:** 1 Department of Physics and Astronomy, University of Denver, Denver, CO, USA; 2 Department of Biological Sciences, University of Denver, Denver, CO, USA

## Abstract

The morphogenesis of developing tissues relies on extensive cellular rearrangements in shape, position, and identity. A key process in reshaping tissues is cell intercalation-driven elongation, where epithelial cells align and intercalate along a common axis. Typically, analyses focus on how peripheral cortical forces influence cell shape changes. Less attention is given to how inhomogeneities in internal structures, particularly the nucleus, impact cell shaping. Here, we examine how pulsed contractile and extension dynamics interact with the nucleus in elongating *Drosophila* embryos. Our data show that tightly packed nuclei in apical layers hinder tissue remodeling/oscillatory behaviors. We identify two mechanisms for resolving internuclear tensions: nuclear deformation and dispersion. Embryos with non-deformable nuclei use nuclear dispersion to maintain near-normal extensile rates, while those with non-dispersible nuclei due to microtubule inhibition exhibit disruptions in contractile behaviors. Disrupting both mechanisms leads to severe tissue extension defects and cell extrusion. These findings highlight the critical role of nuclear shape and positioning in topological remodeling of epithelia.

## Teaser

de Leeuw, Budhathoki, and colleagues examine the mechanical limitations that nuclear volumes impose on epithelial remodeling in *Drosophila*. They identify two primary mechanisms—nuclear dispersion and nuclear deformation—that permit the resolution of internuclear tensions. Failures in both pathways lead to epithelial extrusion.

## Introduction

Epithelial tissues are fundamental building blocks for the construction of many complex animal body plans. These tissues possess an interesting duality of needing to reshape and renew cellular components while maintaining their essential barrier function (Collinet et al., 2015; Loerke and Blankenship, 2020; Martin et al., 2010; Paré and Zallen, 2020; Zallen and Blankenship, 2008). Cell shaping processes are therefore carefully coordinated to permit the emergence of new morphologies while conserving adhesive properties. In the simplest epithelial arrangements, often found in a variety of developmental processes, a monolayer of tightly packed columnar cells is assembled and then shaped. Body axis elongation in the early *Drosophila* embryo is one such example during which oriented intercalations of epithelial cells are driven by the continuous remodeling of cell junctions creating new cellular topologies (Bertet et al., 2004; Blankenship et al., 2006; Irvine and Wieschaus, 1994; Jewett et al., 2017; Rauzi et al., 2010). During this process of tissue extension, the intercalating cells must shift cellular volumes to enter into new neighbor–neighbor relationships, thus directing tissue extension along the anterior–posterior (AP) axis.

The driving of large-scale changes in tissue morphology by small-scale changes in cell size and shape has long invited comparisons to inorganic materials such as foams and colloidal glasses, where the size and relative packing of constituent components produce differential physical properties in the material at large (Bi et al., 2014; Mongera et al., 2018; Weaire and Rivier, 2009; Zallen and Zallen, 2004). While these conceptual parallels have aided in producing successful theoretical descriptions of the material characteristics of tissues, especially in equilibrium conditions, the analogy between living cells and soap bubbles or colloidal suspensions does not fully capture the complexities of cellular life. While the unit cells of inert materials are typically homogenous, living cells are themselves filled with organelles of varying sizes and stiffnesses. Although most organelles are small enough to have a limited effect on whole-cell material properties, the largest organelle, the nucleus, can occupy a significant fraction of the cell volume in a variety of cell types (Mukherjee et al., 2016; Webster et al., 2009). In such cases, the size and stiffness of nuclei may limit the range of surface morphologies achievable by cell boundaries present in near-nuclear vicinities (Bone and Starr, 2016; Cho et al., 2017). To examine this in more detail, and in a model columnar epithelium, we here investigate how nuclear behaviors impact cell topologies during the convergent extension movements that occur in the early *Drosophila* germband.

Indeed, there has been a growing appreciation of the various impacts that nuclei have on tissue-changing processes. During cell migration, nuclear size and stiffness can fundamentally alter migration dynamics in both 2D and 3D spaces (Calero-Cuenca et al., 2018; Friedl et al., 2011; Heo et al., 2020; Kim et al., 2014). Conversely, the weakening of nuclear stiffness during migration can lead to damage to the genetic material it houses (Raab et al., 2016; Shah et al., 2021). The positioning of nuclei in these systems, as well as other tissue-types, has also been shown to be an important component by which cellular functions and homeostasis are maintained. In hyp7 cells of *C. elegans*, a precursor to the hypodermal syncytium, nuclear migration is regulated by lamin-mediated recruitment of linker proteins that in turn activate regulatory components of dynein for microtubule (MT)-based transport (Fridolfsson et al., 2010; Lee et al., 2002). Similarly, during eye development in *Drosophila*, apical migration of nuclei is critical for photoreceptor morphogenesis and is driven by MT and lamin-dependent mechanisms (Kracklauer et al., 2007; Mosley-Bishop et al., 1999; Patterson et al., 2004). Neuroepithelia often show cell-cycle-regulated migration of nuclei and cell division that are again linked to MT- and actomyosin-generated forces during the process of interkinetic migration (Del Bene et al., 2008; Gambello et al., 2003; Ge et al., 2010; Hu et al., 2013; Kosodo et al., 2011; Leung et al., 2011; Norden et al., 2009; Rujano et al., 2013; Spear and Erickson, 2012; Tsai et al., 2007, 2010; Xie et al., 2007). Finally, disruptions of the systems that maintain nuclear behaviors and shapes have been linked to diseased states such as EDMD (Haque et al., 2010; Zhang et al., 2007), Hutchinson Gilford Progeria Syndrome (Kandert et al., 2007), and dilated cardiomyopathies (Nikolova et al., 2004).

In the developmental context of the *Drosophila* embryo, how nuclei respond to the extensive cell shape changes that occur during gastrulation has been unclear. Myosin II is the major contractile force-generating protein present at these stages and is canonically associated with adherens junctions and apicomedial regions where it mediates oscillatory cycles of cell contraction and relaxation (Bertet et al., 2004; Fernandez-Gonzalez and Zallen, 2011; Rauzi et al., 2010; Sawyer et al., 2011; Vanderleest et al., 2018, 2024; Zallen and Wieschaus, 2004). However, whether nuclear mechanical properties respond and/or affect these dynamics has been unclear. Here, we identify two parallel pathways—nuclear deformation and nuclear dispersion—by which nuclei support cellular remodeling while still maintaining regularity in cell and tissue dimensions. We quantify the contributions of each pathway to the interfacial remodeling that directs intercalation and tissue elongation, as well as the barrier that they impose on cell shapes and actomyosin contractile pulses. We also observe a potential link between higher internuclear tensions and the forced exclusion of cells from apical layers.

## Results

### Nuclei are tightly packed in a common apical plane at the onset of cell intercalation

**Figure 1.**
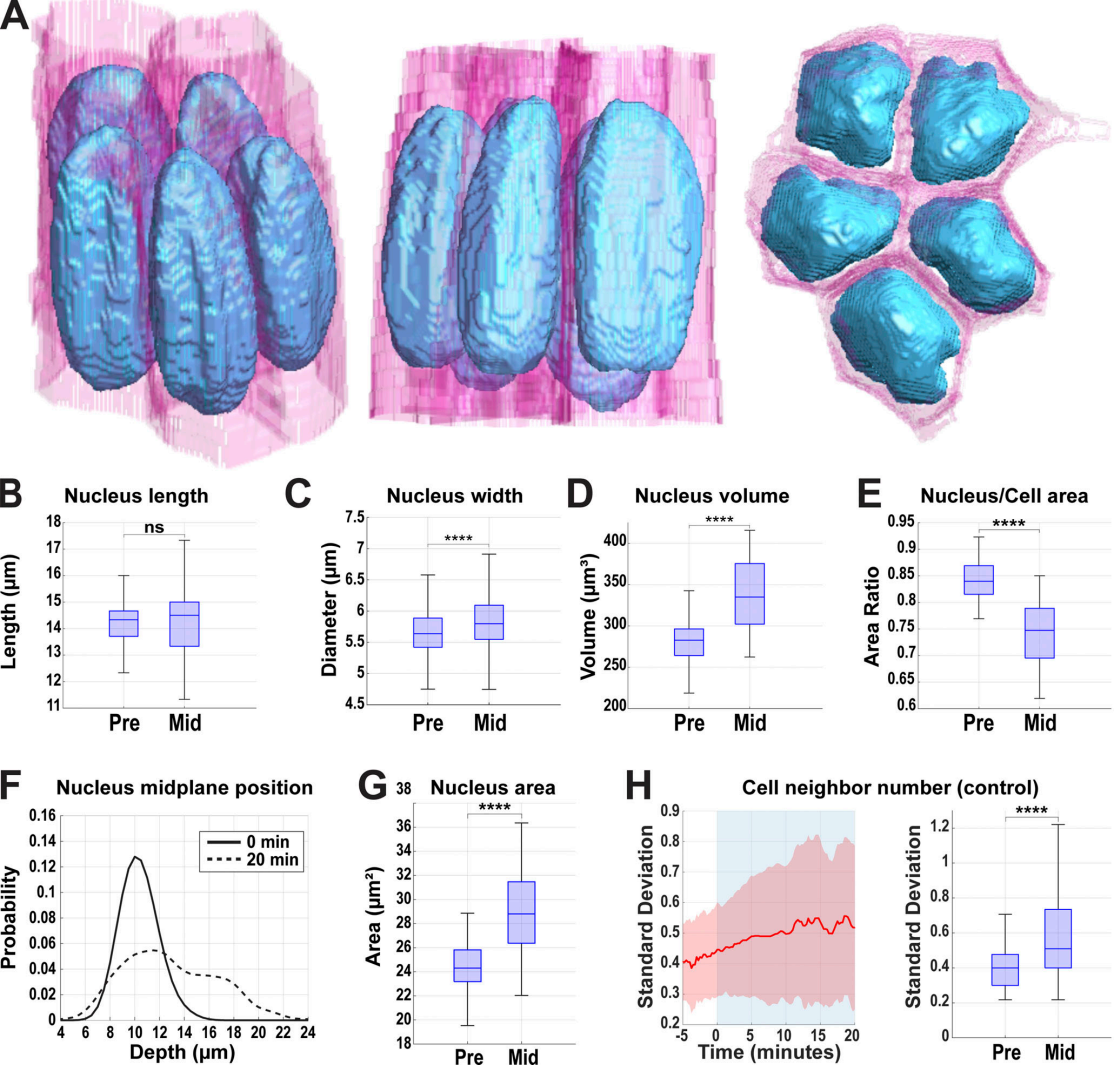
**Nuclei are arranged in a common apical plane at the start of tissue extension. (A)** 3D reconstruction of cell membranes (pink) and nuclei (blue) from live imaging data. Slanted view highlighting nuclear surfaces (left), side view showing close apposition of nuclei in the packed epithelium at the start of tissue extension (mid), and top view showing how efficiently nuclei are packed in the available cytoplasm (right) (related to Video 1). **(B)** Measurement of nucleus length pre- (−5 min) and mid- (+20 min) GBE. **(C)** Comparison of nucleus width at the midplane at pre- (−5 min) and mid- (+20 min) GBE; *n* = 558 for pre- and 332 for mid-GBE. **(D)** Comparison of nucleus volume before (pre = −5 min) and after (mid = +20 min) the onset of GBE. **(E)** The ratio of nucleus area to cell area at the nuclear midplane compared before (pre = −5 min) and after (mid = +20 min) the onset of GBE. For B, D, and E, *n* = 388 nuclei. **(F)** Probability of absolute position of nuclear midplane along the apical–basal axis at 0 and 20 min of GBE; *n* = 82 nuclei for 0 min and 47 nuclei for 20 min from k = 1 embryo. **(G)** Area of nuclei at their midplane pre- (−5 min) and mid- (+20 min) GBE; *n* = 388 nuclei. **(H)** Median SD of cell neighbor number from 5 min before to 20 min after the onset of GBE (left) and comparing SD of cell neighbor number at two time points, 5 min prior (pre) to the onset of GBE and 20 min mid-GBE initiation (right); *n* = 1,131 cells. 0 min indicates the onset of GBE and GBE is indicated by blue background. For B–G (except F), the data were collected from k = 5 embryos. The measured *n* values are regardless of timepoints where not indicated. For B, D, E, and G, statistical significance was calculated using Student’s *t* test. For C and H, statistical significance was calculated using the Mann–Whitney U-test. ns = not significant, ****P < 0.0001.

**Figure S1.**
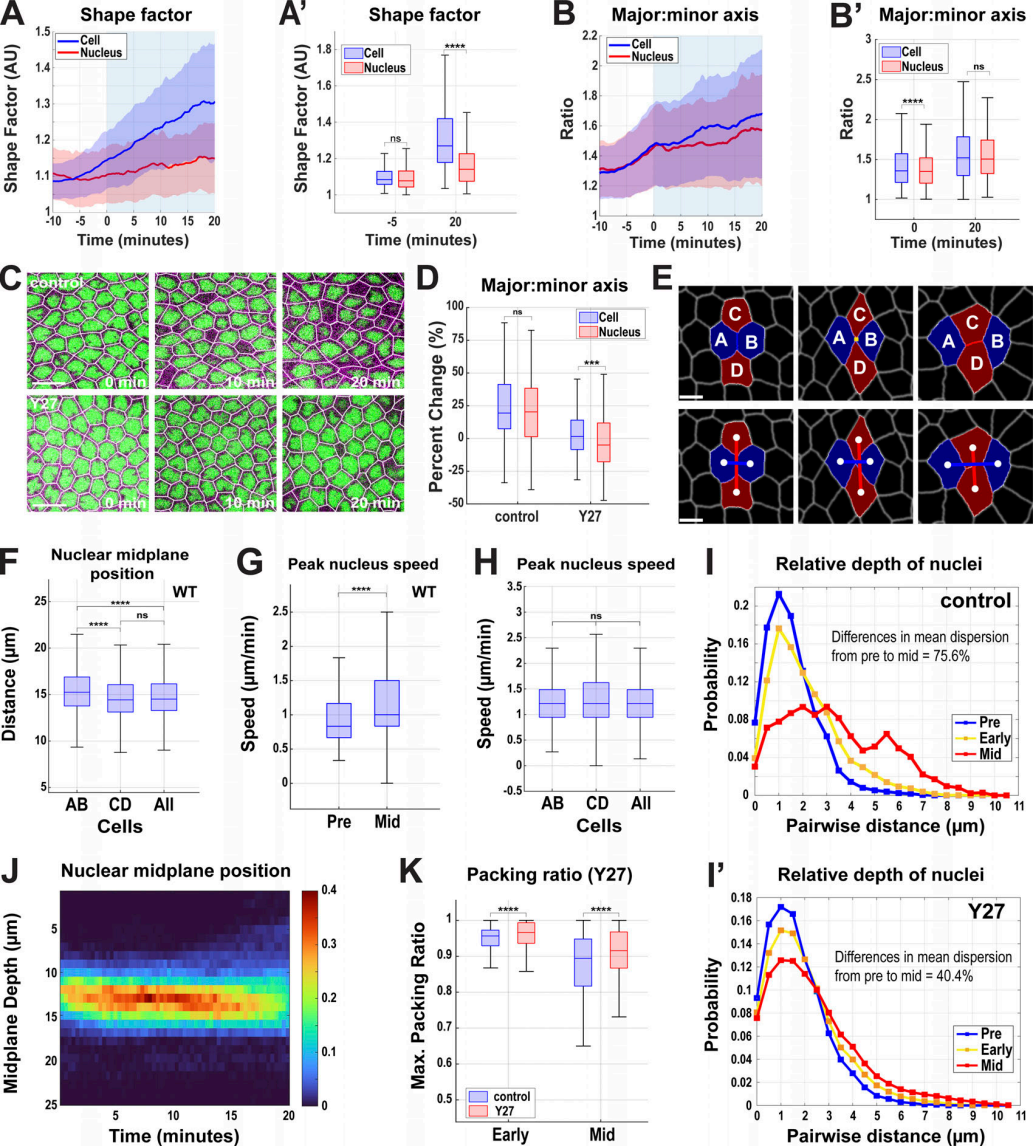
**Quantitation of cell and nuclear behaviors in control and myosin disrupted epithelia. (A and A′)** Shape factor at the nuclear midplane for cell (blue) and nucleus (red) from 10 min prior to 20 min mid-GBE in wildtype embryos and a box chart showing the comparisons at −5 and 20 min of GBE in A′. **(B)** Ratio of major to minor axis length for cell (blue) and nucleus (red, same as in Fig. 2 B) at nuclear midplane from 10 min prior to 20 min mid-GBE in wildtype embryos. **(B′)** Major:minor axis ration at the nuclear midplane for cell (blue) and nucleus (red) at 0 and 20 min mid-GBE in wildtype embryos. For A–B′, *n* = 605 cells and nuclei from k = 5 embryos. For A and B, light blue shading indicates GBE. Error envelopes indicate SD. **(C)** Still images showing cell deformation is required for nuclear deformation and dispersion indicated by fairly round nuclei present in Y-27632–injected (Y27) embryos compared to vehicle-injected (control) embryos. **(D)** Comparison of the major:minor axis percent change at nuclear midplane for cell (blue) and nucleus (red) between control and Y27 embryos. The negative percent change indicates a decrease in the axis ratio and the positive percent change indicates an increase in the axis ratio; *n* = 186 cell and nuclei for control and *n* = 236 cell and nuclei for Y27 from k = 3 embryos for each background. **(E)** Example of T1 transition with a color overlay of T1 interface (blue) shared by cells A and B, T2 vertex (yellow) shared by cells A–D, and T3 interface (red) shared by cells C and D (top). Bottom panels show the centroid (white) distance between AB (blue) and CD (red) cells. **(F)** Absolute nuclear midplane positions in AB, CD, and All cells. **(G)** Peak velocity with which nuclei move in apical–basal direction measured pre- (−5 to 0 min) and mid- (+15 to +20 min) GBE in wildtype embryos; *n* = 213 nuclei for pre- and *n* = 512 for mid-GBE from k = 3 embryos. **(H)** Maximum apical–basal speed of nuclei in AB, CD, and All cells. For F and H, *n* = 36 for AB and CD cells and *n* = 82 for All cells from k = 1 embryo. **(I and I′)** Comparison of probability change in relative nuclear position at pre- (−5 to 0 min, blue), early (0–10 min, yellow) and mid- (15–20 min, red) GBE in control and Y27 embryos; *n* = 3,339 nucleus pairs for control and *n* = 5,565 nucleus pairs for Y27 embryos from k = 3 embryos for each background. **(J)** Heatmap showing apical–basal distribution of nuclei (absolute midplane position) over the course of GBE in Y27 embryos. Color bar indicates the probability of nuclear midplane position; *n* = 1,097 nuclei from k = 3 embryos. **(K)** Maximum packing ratio achieved by clusters of nuclei as measured by the dispersion metric at early (0 min) and mid- (+20 min) GBE; for control, *n* = 370 clusters of cells for early and 336 clusters of cells for the mid-GBE, for Y27, *n* = 803 clusters of cells from early and 870 clusters of cells from mid-GBE from k = 3 embryos for each background. The measured *n* values are regardless of time points where not indicated. For C and E, scale bar = 10 and 5 μm, respectively. Statistical significance was calculated using the Mann–Whitney U-test. ns = not significant, ***P < 0.001, ****P < 0.0001.

We first examined the size, positioning, and morphologies of nuclei in the *Drosophila* epithelium just prior to the onset of tissue extension. Segmentation and analysis of nuclear shapes (as marked by RFP:NLS) show that nuclei at these stages are elongated and highly featured ovoid structures that are approximately twice as long they are wide and possess mean volumes of about 280 µm^3^ (Fig. 1, A–D and Video 1). Importantly, nuclei occupy a large fraction of the cell cross-sectional area at their widest point and closely approach cell boundaries, on average occupying 84% of the available cross-sectional area in the cell (Fig. 1, A and E). Despite being highly crowded, prior to the onset of intercalation nuclei are preferentially positioned at a common depth in cells, with the nuclear midplanes positioned around 10 µm below the apical surface (Fig. 1 F). The prospect for overcrowding within a common apical–basal plane only becomes more apparent over the course of tissue extension (also known as germband extension, or GBE)—while nuclei retain a relatively fixed length (Fig. 1 B), on average their volume and maximum cross-sectional area both increase by 15–20% over pre-GBE levels (Fig. 1, D and G; P < 0.0001, *n* = 388 nuclei). At the cellular level, cells adopt increasingly more extreme shapes and topologies as intercalation proceeds, with cell shape factors (a metric describing the degree to which an object approaches a circular shape) increasing and topologies becoming increasingly disordered (Fig. 1 H and Fig. S1, A–B′) (Blanchard et al., 2009; Farhadifar et al., 2007; Vanderleest et al., 2022; Zallen and Zallen, 2004). These data suggest that as cells undergo the extensive reshaping of morphologies necessary for successful tissue extension, mechanisms must exist that allow for the accommodation of bulky nuclei.

**Video 1.** **Nuclei are highly featured and elongated structures in the early germband epithelium.** 3D segmentation of nuclei (RFP:NLS) and cell outlines (Spider:GFP) from control embryos at early germband extension. The movie displays two rotations of segmented cells and nuclei as shown in Fig. 1 A. 30 frames/second. Scale bar = 5 μm.

### Nuclei possess a limited ability to deform to match cell topologies

**Figure 2.**
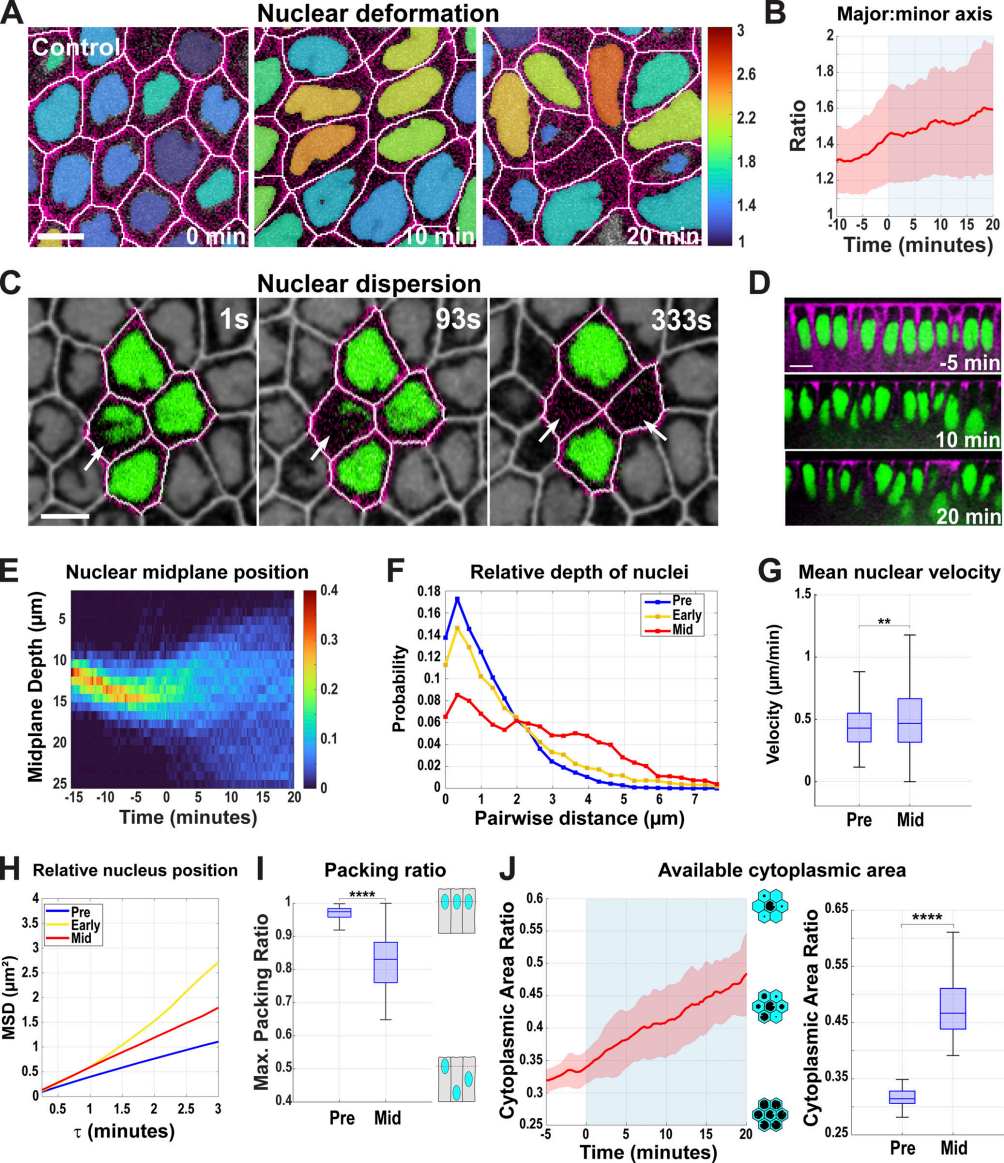
**Nuclear deformation and dispersion during tissue extension. (A)** Still images from a time-lapse movie of embryo expressing nuclear (NLS: RFP) and cell outline marker (Spider:GFP) showing nuclear deformation as GBE proceeds (related to Video 2). Nuclei are color-coded according to their major-to-minor axis ratio in the XY plane. Image slice from ∼10 µm below the apical surface. **(B)** Nuclear shape changes represented by ratio of major to minor axis at the nuclear midplane from pre- to mid-GBE (−10 to +20 min); *n* = 605 nuclei from k = 5 embryos. **(C)** Still frames showing nuclear dispersion indicated by disappearance of nuclei from the given imaging plane (marked by arrows) during a T1 cell intercalation event (related to Video 3). Image slice from ∼8 µm below the apical surface. **(D)** Cells with their nuclei in apical–basal axis at −5 min (top), 10 min (center), and 20 min (bottom) of GBE onset showing nuclei undergo dispersion relative to cells as GBE proceeds. **(E)** Heatmap showing wide distribution of nuclei in apical–basal axis (absolute midplane position) as GBE progresses. Color bar indicates the probability of nuclear midplane position; *n* = 606 nuclei from k = 3 embryos. **(F)** Probability distribution of nuclei along apical–basal axis relative to their neighboring nuclei in pre- (−5 to 0 min, blue), early (0–10 min, yellow), and mid- (15–20 min, red) GBE; *n* = 16225 pairs of nuclei from k = 3 embryos. **(G)** Mean nuclear velocities at pre- (−10 to 0 min) and mid- (15–20 min) GBE, *n* = 213 nuclei for pre- and *n* = 512 for mid-GBE from k = 3. **(H)** MSD (pre = −5 to 0 min, early = 0–10 min, and mid = 15–20 min); *n* = 16,225 pairs of nuclei from k = 3 embryos. **(I)** Maximum packing ratio achieved by clusters of nuclei as measured by the dispersion metric (see Materials and methods) at pre- (−5 min) and mid- (+20 min) GBE; *n* = 152 clusters of cells from k = 3 embryos. Schematics on the top right exemplifies ratio of 1.0 when nuclei (blue) are in the same apical–basal plane (red dotted line) and the ratio decreases as nuclei spread across different planes (bottom right). **(J)** Average cytoplasmic area (ACA) ratio available in cell clusters (schematics depict cytoplasmic areas in blue and nuclei areas in black) at the level of the nuclear midplane of a given central cell during GBE (left) and box and whisker plot showing significance if ACA metric at 5 min before (pre) and 20 min (mid) after the onset of GBE (right); *n* = 206 clusters of cells from k = 3 embryos. For B and J, light blue background indicates GBE. Error envelopes indicate SD. For A and C, scale bar = 5 µm and for D, scale bar = 10 μm. The measured *n* values are regardless of timepoints where not indicated. For I, statistical significance was calculated using Student’s *t* test. For G and J, statistical significance was calculated using the Mann–Whitney U-test. **P < 0.01, ****P < 0.0001.

While the cross-sectional profiles of nuclei typically display more rounded morphologies as compared with polygonal cell outlines, a close examination shows that nuclei conform, to some extent, to the shape of their germband epithelial cell (Fig. 1 A). We were therefore interested in whether nuclear shapes also become more anisotropic as cells undergo intercalary movements. Indeed, nuclei deform to accommodate changes in cell dimensions during tissue extension, with planar nuclear elongation measurements also increasing as GBE proceeds (Fig. 2, A and B; and Fig. S1, A–B′; and Video 2). This deformation is dependent on cortical actomyosin force generation, as they remain more equally dimensioned in embryos that have been injected with the Rho kinase inhibitor Y-27632 (Fig. S1, C and D). Measurements of shape factor show that, while epithelial cells and nuclei have almost identical shape factors just prior to the onset of intercalation, during tissue extension the median shape factor for cells increases by a much greater extent than for nuclei (Fig. S1, A and A′). Nuclei have a lesser degree of anisotropic deformation than cells, and thus shape factor and aspect ratio measurements of nuclei do not rise to the same extent as cell geometries (Fig. S1, A–B′). Nuclei also maintain a relatively constant height, suggesting that apical–basal elongation of the nuclei does not occur in response to increased lateral compression as cells change shape (Fig. 1 B). These data suggested the possibility that another pathway, other than deformation, may exist to relieve nuclear crowding during tissue extension.

**Video 2.** **Nuclei deform over the course of GBE to accommodate cell shape changes.** The movie shows control nuclei labeled by the ratio of their major and minor axis lengths, as seen in Fig. 2 A. More rounded nuclei are in blue, while elongated/deformed nuclei appear in hotter colors (orange or red). Movies were acquired at a rate of 15 s per frame and displayed at 12 frames per second. Scale bar = 10 μm.

### Nuclear dispersion decreases crowding and frees cytoplasmic space for morphogenesis

Previous work has demonstrated that epithelia often possess nuclear positioning pathways essential for migratory or morphogenetic behaviors (Lee and Norden, 2013; Starr and Fridolfsson, 2010). Indeed, a visual inspection of nuclear occupancy shows that nuclei begin moving into other apical–basal layers as cell intercalation occurs (Fig. 1 F; and Fig. 2, C and D; and Video 3). Intercalary behaviors are topology-driven processes in which AP interfaces between anterior and posterior cells (also known at T1 interfaces) contract to form a single vertex between four cells (or more in rosette configurations) followed by the extension of a new DV interface between the newly neighboring dorsal and ventral cells (identified as T3 interfaces) (Fig. S1 E) (Bertet et al., 2004; Irvine and Wieschaus, 1994; Yu and Fernandez-Gonzalez, 2016). We observed that as cells progress through a sample T1–T3 neighbor exchange, the nuclei that shared a common T1 interface move to basal regions (marked by arrows in Fig. 2 C). While which cells in a T1 configuration shift their nuclei basally varies (Fig. S1 F), a broad dispersal of nuclei is seen in global measurements of nuclear positioning during GBE. This dispersal occurs specifically at the onset of intercalation and involves the movement of nuclei away from their original shared apical plane toward a range of depths (Fig. 2, D–F).

**Video 3.** **Nuclei disperse to different apical–basal planes during tissue extension.** Video displays nuclei (at a given plane) from a control embryo labeled by midplane depth from the apical surface. At the beginning of GBE, nuclei are located in a common plane (yellow) color; as intercalation initiates, nuclei disperse in apical (hotter, red colors) and basal directions (cooler, blue colors). Nuclei that move completely out of the imaging plane are not displayed from that time point forward. Images in videos were acquired at a rate of 15 s per frame and displayed at 12 frames per second. Scale bar = 10 μm.

Indeed, apical–basal nuclear displacements increase throughout GBE. Average nuclear velocities increase during GBE, with peak velocities as high as 2.5 µm/min (Fig. 2 G and Fig. S1 G). The peak nuclear velocity is comparable in all cells involved in T1 configuration (Fig. S1 H). Mean squared displacement (MSD)–based analysis of nuclear trajectories also demonstrates the characteristic upward curvature of an actively driven process during early germband extension (Fig. 2 H). This dispersion significantly reduces the maximum packing of nuclei experienced by the tissue at any given depth, with peak nuclear densities being reduced by 21% over the first 20 min of tissue extension (Fig. 2 I; P < 0.0001, *n* = 152 cell clusters). Importantly, this reduction of nuclei packed in a common plane nearly doubles the available cytoplasmic area (the cross-sectional cell area in a given plane that is not occupied by nuclei, see Materials and methods), potentially permitting cell centroid displacements necessary for tissue flow and topological remodeling (Fig. 2 J). Similar to nuclear deformation, dispersion of nuclei is reduced in embryos that do not undergo intercalation due to disrupted contractile force generation and the maximum packing of nuclei is not reduced to the same level as compared to the vehicle control (Y-27632 Rho kinase inhibitor injection, Fig. S1, C and I–K). Thus, our results reveal two primary mechanisms, nuclear deformation and nuclear dispersion, by which nuclei may adapt to the cell shape changes that drive tissue extension in the early embryonic epithelium.

### Nuclear deformation is essential for efficient cell packing and tissue extension

**Figure 3.**
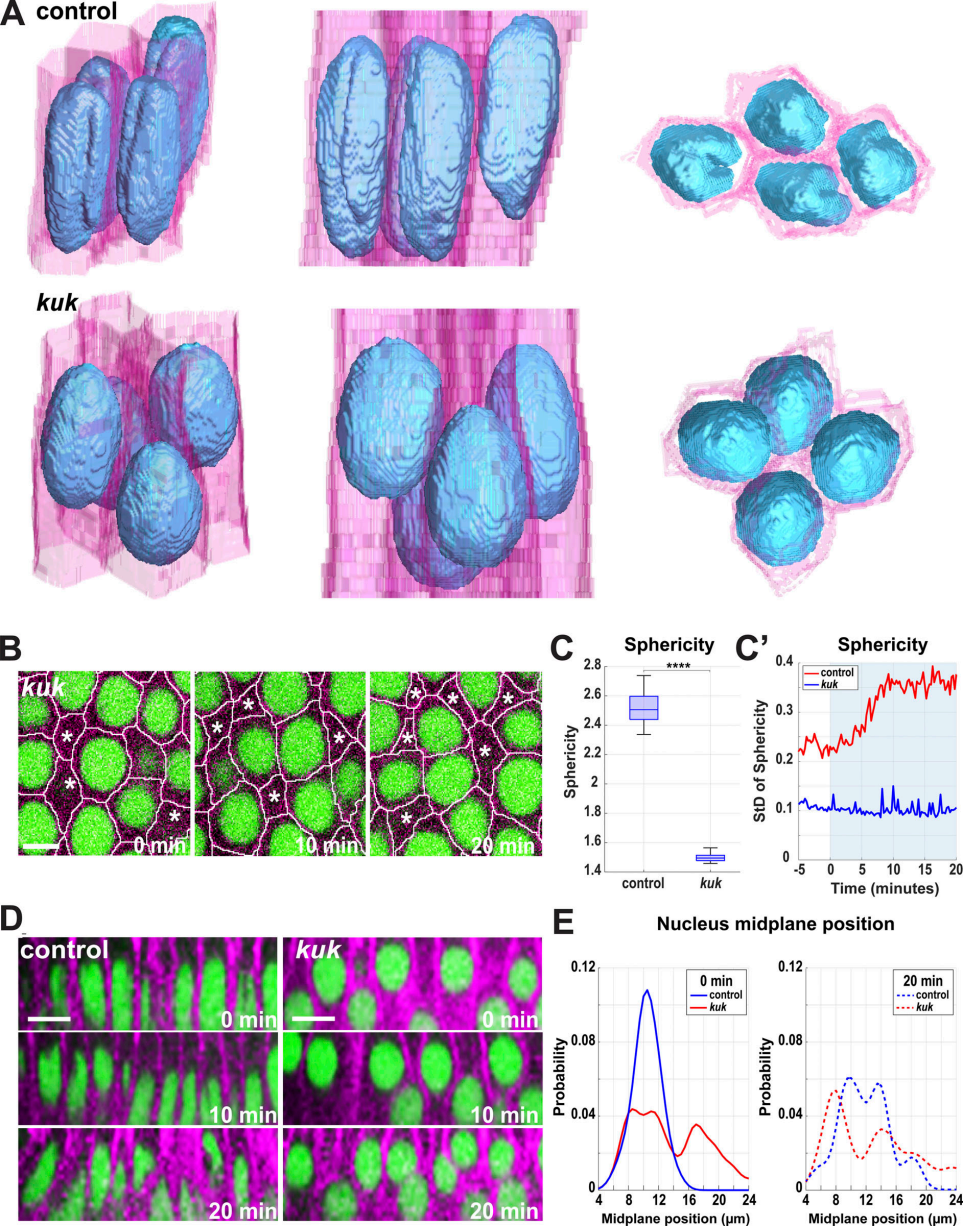
**Tissue and cellular architecture in embryos with non-deformable nuclei. (A)** 3D reconstruction of cell membranes (pink) and nuclei (blue) from live imaging data of control (*luciferase* shRNA, top panel) and *kuk* shRNA (bottom panel) embryos. The slanted view shows *kuk* embryos with round deformation-resistant nuclei and smoother nuclear surfaces (left), side view reveals that *kuk* nuclei accommodate themselves in different apical–basal planes and partially deform cell shapes (mid), and the top view shows nuclear packing and potential overlaps (right) compared with the control (related to Video 5). **(B)** Still images from *kuk* embryos showing greater dispersion of nuclei, indicated by cell cross-sections without nuclei (marked by asterisks). These rounded nuclei resist deformation even while cell shapes are becoming increasingly irregular. Image slice from ∼11 µm below the apical surface. **(C)** Measurement of nuclear sphericity in control and *kuk* embryos during GBE. **(C′)** SD of nucleus sphericity for control (red) and *kuk* (dark blue) embryos showing *kuk* nuclei resisting deformation throughout GBE. The light blue background indicates GBE. For C and C′, *n* = 605 control nuclei and *n* = 876 *kuk* nuclei from k = 3 embryos for each background. **(D)** Orthogonal view of control (*luciferase*) and *kuk* nuclei showing greater dispersion in *kuk* embryos. **(E)** Probability of absolute position of nuclear midplane along the apical–basal axis at 0 and 20 min of GBE for control (*luciferase*) and *kuk* embryos; for control, *n* = 116 nuclei for 0 min and *n* = 81 nuclei for 20 min, for *kuk*, *n* = 81 for 0 min and *n* = 79 for 20 min from k = 1 embryo for each background. For B and D, scale bar = 5 and 10 μm, respectively. Statistical significance in C was calculated using Student’s *t* test. ****P < 0.0001.

**Figure S2.**
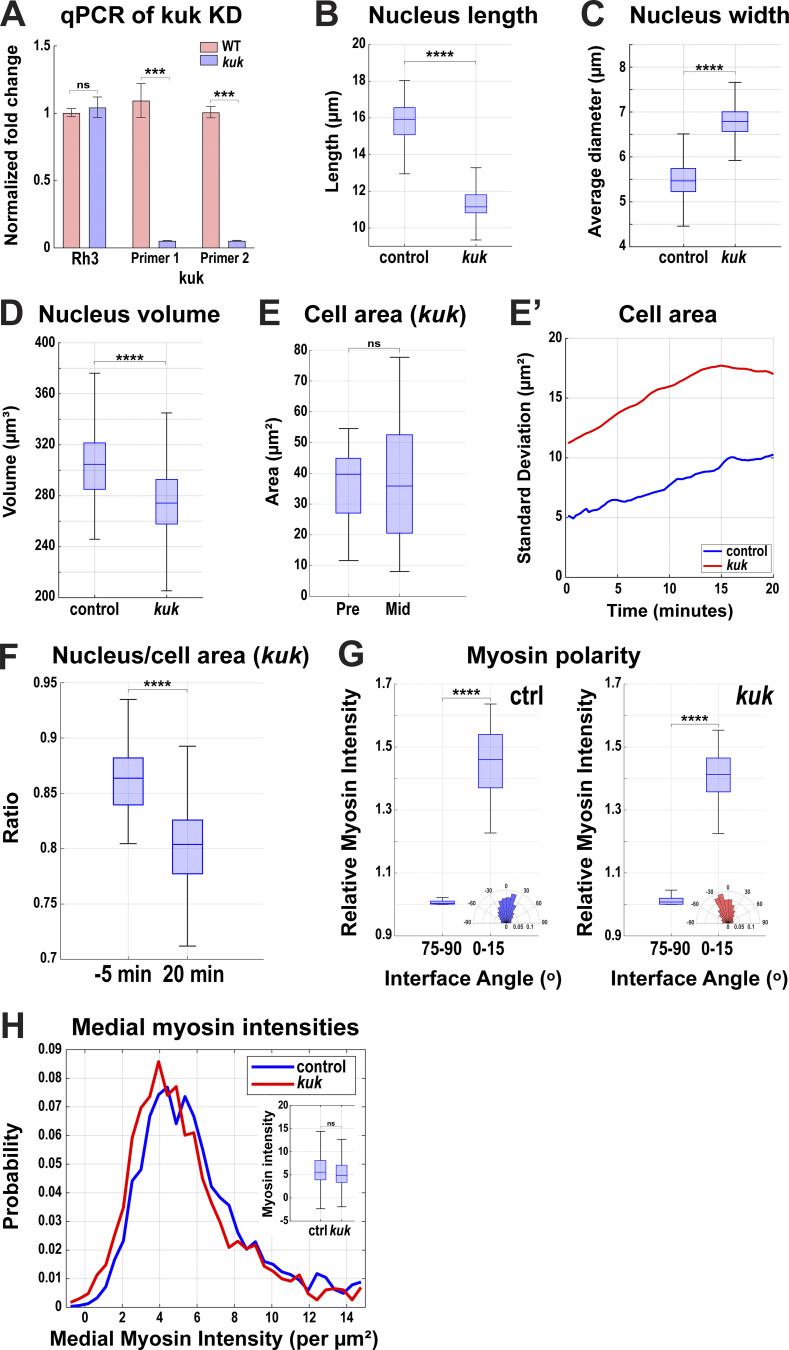
**Nuclear measurements in *kuk* disrupted embryos. (A)** Fold change of expression of Rh3 and kuk using two different primers obtained by normalizing with sqh (positive) control. **(B)** Comparison of 3D long-axis length of nuclei in control (*luciferase* shRNA) and *kuk* embryos. **(C)** Comparing nucleus width at nuclear midplane for control and *kuk* embryos; *n* = 9,611 nuclei and *n* = 315 nuclei for *kuk* from k = 3 embryos for each background. **(D)** Comparison of nuclear volume in control and *kuk* embryos. For B and D, *n* = 961 nuclei for control and *n* = 580 nuclei for *kuk* from k = 3 embryos for each background. **(E)** Cell area measured at apical region (z-depth = 7 μm from the apical surface) in *kuk* embryos demonstrating increased variation in cell areas at the later time point of GBE indicated by wider whiskers at 20 min; *n* = 285 cells from k = 3 embryos. **(E′)** Comparison of SD of cell area (z-depth = 7 μm from apical surface) in control and *kuk* embryos from the onset (0 min) to 20 min after the onset of GBE; *n* = 961 cells for control and *n* = 580 for *kuk* from k = 3 embryos. **(F)** Ratio of nucleus area to cell area at the nuclear midplane in *kuk* embryos compared at −5 min and +20 min of GBE; *n* = 285 cells and nuclei from k = 3 embryos. **(G)** Relative myosin intensity at horizontal (75–90° interface angle) and vertical (0–15° interface angle) interfaces showing myosin polarity in control and *kuk* embryos; *n* = 2,723 interfaces for control, *n* = 2,827 interfaces for *kuk* from k = 3 embryos for each background. Insets show rose plots showing directionality of contracting interface; *n* = 1,601 interfaces for control and *n* = 1,387 interfaces for *kuk* from k = 3 for each background. **(H)** Probability distribution of medial myosin intensities (per square microns) in wildtype and *kuk* embryos. The box chart in the inset shows no significant changes in myosin intensities due to *kuk* disruption; *n* = 1,011 cells for wildtype and *n* = 882 cells for *kuk* from k = 3 embryos for each background. The measured *n* values are regardless of time points where not indicated. For F, statistical significance was calculated using Student’s *t* test. For A–C, H, and I, statistical significance was calculated using the Mann–Whitney U-test. ns = not significant, ***P < 0.001, ****P < 0.0001.

**Figure 4.**
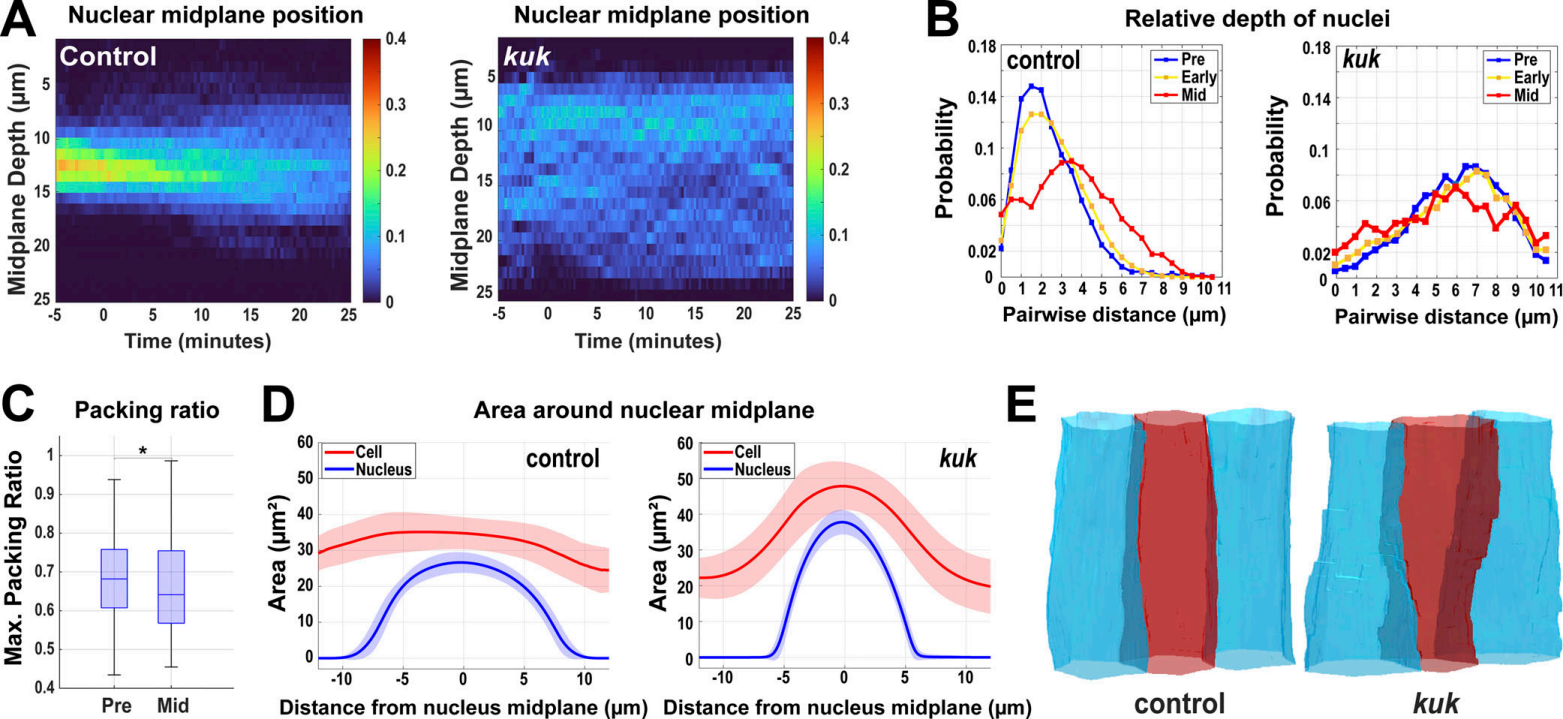
**Enhanced dispersion of nuclei in the absence of the deformation pathway. (A)** Heatmap showing the apical–basal distribution of nuclei (absolute midplane position) for control (*luciferase* shRNA) and *kuk* shRNA embryos. Color bar indicates the probability of nuclear midplane position; *n* = 888 for control and *n* = 929 nuclei from k = 3 embryos for each background. **(B)** Probability distribution of relative depth of nuclei at pre- (−5 to 0 min, blue), early (0–10 min, yellow), and mid- (15–20 min, red) GBE for control (*luciferase*) and *kuk* embryos; *n* = 21,303 pairs of nuclei for control (*luciferase*) and *n* = 13,857 pairs of nuclei for *kuk* from k = 3 embryos for each background. **(C)** Maximum packing ratio of nuclear clusters at pre- (−5 min) and mid- (+20 min) GBE for *kuk* embryos; *n* = 580 clusters of cells from k = 3 embryos. **(D)** Nuclear position (nucleus area, blue) correlates with cell bulging (indicated by increased cell area, red). Cell deformation is enhanced by the deformation-resistant nuclei (*kuk*). Negative x-axis values indicate apical regions above nuclear midplane and the positive values indicate the basal regions; *n* = 317 control and *n* = 357 *kuk* cell-nuclei pairs from k = 3 embryos for each background. Error envelopes indicate SD. **(E)** Side views of 3D cell reconstruction from time-lapse movie of control (*luciferase*) and *kuk* embryos depicting higher degree of cell deformation in cells with non-deformable nuclei (*kuk*). The measured *n* values are regardless of timepoints. For C, statistical significance was calculated using the Mann–Whitney U-test. *P < 0.05.

As the above results suggest how nuclei may respond to changes in cell shape, we next wanted to examine the effects of disrupting the nuclear deformation pathway on epithelial behaviors and tissue extension. To do so, we analyzed epithelia that have “non-deformable” nuclei through inhibition of the *kugelkern* (*kuk*) gene (Video 4), which encodes a lamin-like protein implicated in developmental plasticity of nuclei (Brandt et al., 2006; Hampoelz et al., 2011). We verified *kuk* disruption by comparing the nuclear phenotypic similarity with previous studies on *kuk* (Brandt et al., 2006; Hampoelz et al., 2011; Pilot et al., 2006) (Fig. 3, A and B), as well as by quantitative PCR (qPCR) (∼95% decrease; Fig. S2 A). Nuclei in these embryos are highly spherical and appear to lack the many small furrows and depressions in their surfaces that control nuclei possess (Fig. 3, A, C, and C′; and Video 5). While control embryos have nuclear sphericity measurements around 2.5 (see Materials and methods), *kuk* nuclei are nearly equally dimensioned in all axes with a sphericity around 1.5 on average (Fig. 3 C). *kuk* nuclei are ∼1.4 times shorter (Fig. S2 B) but are 24% wider at the nuclear midplane (Fig. S2 C). They have a slightly decreased volume (12%) as compared with control nuclei (Fig. S2 D) and also appear to undergo next to no deformation over the course of tissue extension (Fig. 3, B and C′; P < 0.0001, *n* = 605 control, 876 *kuk* nuclei). Intriguingly, this disruption of nuclear deformation led to a greater, and earlier dispersion of nuclei, with fewer nuclei occupying common apical–basal planes (Pilot et al., 2006) (Fig. 3, A, B, D, and E; and Fig. 4, A and B). This inability to deform nuclei also produced cells that appear to be challenged in the degree to which they could regularly pack together, with cross-sectional areas along the apical–basal axis becoming highly variable (Pilot et al., 2006) (Fig. S2, E and E′). The *kuk* nuclei occupied 86% (compared to 84% for WT) of the cell area at the nuclear midplane, which stayed relatively constant throughout GBE and the maximum packing of nuclei was also maintained between 60% and 70% (Fig. S2 F and Fig. 4 C). These data also revealed the local neighborhood influences of nuclei, as the presence of a deformation-resistant nucleus clearly affected the cellular dimensions of not only its own cell but also those of neighboring epithelial cells (Fig. 4, D and E). Importantly, these behaviors permit the testing of how well topology-changing contractile movements occur when the deformation pathway is compromised.

**Video 4.** **Deformation-resistant nuclei display enhanced dispersion and sphericity.** Live imaging of embryos expressing RFP:NLS (false-colored green) and Spider:GFP (magenta) in wildtype control (left) and *kuk* disrupted (right) embryo. Note that fewer nuclei are in a common plane in *kuk* embryos. Frames are acquired every 15 s and the movie is played at 20 frames per second. Scale bar = 10 μm.

**Video 5.** ***ku******k***** nuclei are shorter, rounder, and have smoother surfaces.** 3D segmentation of nuclei (RFP:NLS) and cell outlines (Spider:GFP) from control embryos at early germband extension. The movie displays two rotations of segmented cells and nuclei as shown in Fig. 3 A. 30 frames/second. Scale bar = 5 μm.

**Figure 5.**
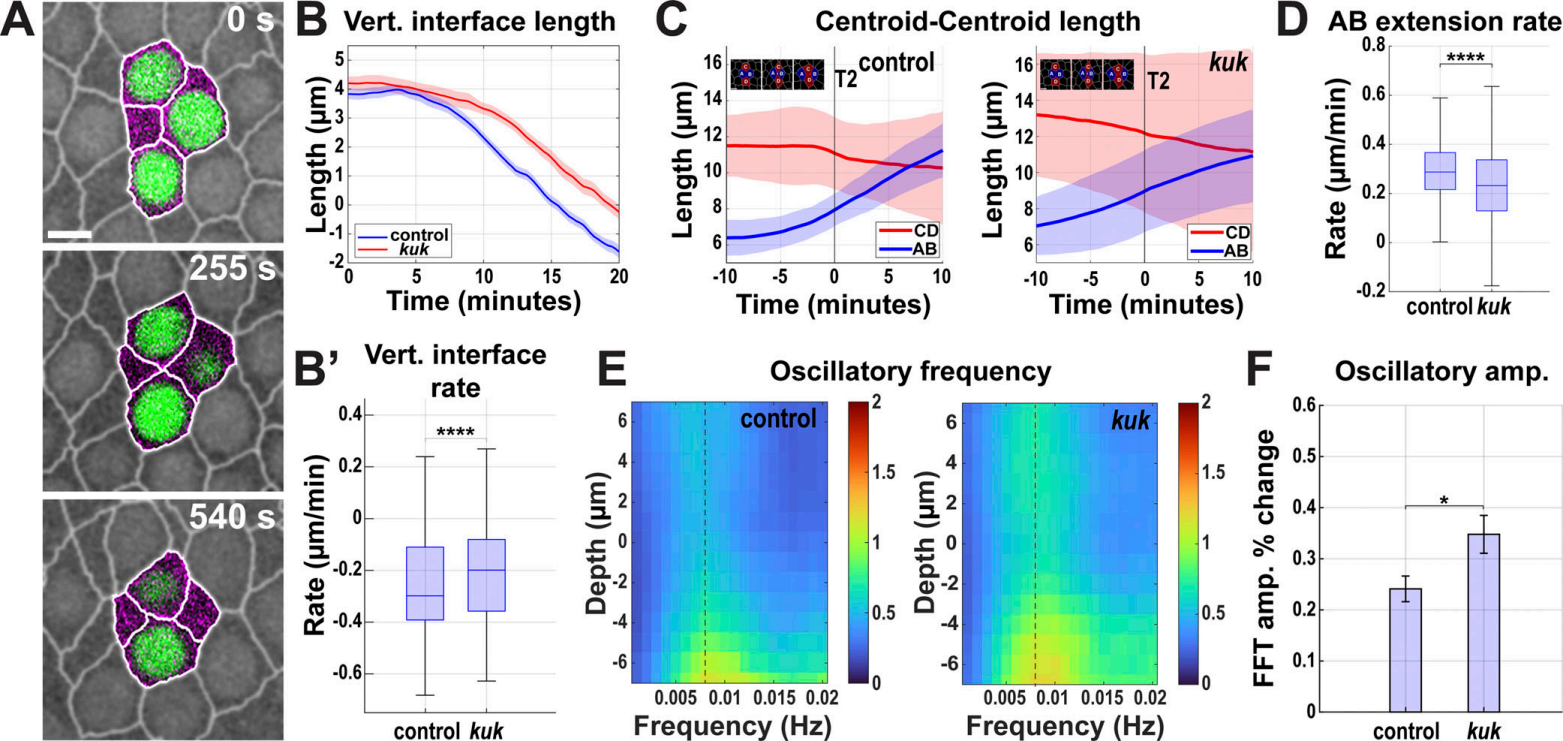
**Contractile pulse and extension dynamics in epithelia with non-deformable nuclei. (A)** Still frames of T1 events from a time-lapse *kuk* movie showing enhanced nuclear dispersion (indicated by the disappearance of nuclei from the given imaging plane at 0 s). Image slice from ∼11 µm below the apical surface. **(B)** Comparing vertical interface lengths of control (*luciferase shRNA*) and *kuk* shRNA during GBE. 0 min indicates the onset of the GBE. Positive lengths indicate T1 interfaces while negative lengths are horizontally extending T3 interfaces. Error envelopes indicate SEM. **(B′)** The rate of vertical interface length change is shown in B′, negative values indicate contraction of vertical interfaces; *n* = 133 interfaces for control (*luciferase*) and *n* = 95 interfaces for *kuk* from k = 3 embryos for each background. **(C)** Centroid distances for AB (blue) and CD (red) cells over the course of 20 min for control (*luciferase*) (left) and *kuk* (right) embryos during GBE indicating cell convergence and extension. Inset shows labeling scheme of AB and CD cells during T1 to T3 transitions. Error envelopes indicate SD. **(D)** Extension rate of AB cells in control (*luciferase*) and *kuk* embryos. For C and D, *n* = 158 for control (*luciferase*) and *n* = 159 transitions for *kuk* from k = 3 embryos for each background. **(E)** FFT analysis of oscillatory dynamics in control (*luciferase*) and *kuk* embryos. The dotted line indicates where the amplitude percent change was calculated in F. Color bar indicates the FFT amplitude in the frequency space. **(F)** FFT amplitude percent change (decrease in cell oscillation amplitude from the nucleus midplane to 4 μm basal to the nucleus midplane) in control (*luciferase*) and *kuk* embryos. For E and F, *n* = 961 cells for control and *n* = 580 cells for *kuk* from k = 3 embryos for each background. Error bars indicate SEM. The measured *n* values are regardless of time points. Scale bar = 5 µm. Statistical significance was calculated using the Mann–Whitney U-test. *P < 0.05, ****P < 0.0001.

To examine this, we measured intercalary dynamics in embryos that possessed non-deformable nuclei. Interestingly, *kuk* embryos still could undergo cell–cell neighbor exchange events (Fig. 5 A), though the rates at which these occur were compromised. The contraction of vertical interfaces that drive intercalation was reduced by ∼33% (P < 0.0001, *n* = 133 control and 95 *kuk* interfaces) as compared with control shRNA embryos (Fig. 5, B and B′). A key to tissue extension is that the changes in cell interface dimensions should lead to the eventual displacement of cell centroids (and bulk cell volume) so as to drive effective tissue elongation and intercalation (Collinet et al., 2015). Similar to the reductions in vertical interface length rates, *kuk* embryos had a moderate 20% reduction in extension as measured by centroid displacements in intercalating cells (measuring the distance separation between the AB cell centroids that share a common T1 interface), indicating slower effective tissue extension (Fig. 5, C and D; and Fig. S1 E; P < 0.0001, 158 control, 159 *kuk* transitions). Myosin polarity, medial myosin intensities, and directionality of contracting interfaces were not compromised by *kuk* inhibition (Fig. S2, G and H). These data suggest that internuclear frictions between non-deformable nuclei inhibit the free flow of the bulk of cell volumes as cells attempt to move in the remodeling epithelium. However, it also appears that the greater dispersion of nuclei in *kuk*-compromised embryos may compensate for the disruption of nuclear deformation, thus allowing interface contraction and extension to occur in only a partially diminished fashion. Finally, we examined the contractile oscillations in cell areas that underlie intercalary behaviors in the germband epithelium (Fernandez-Gonzalez and Zallen, 2011; Rauzi et al., 2010; Sawyer et al., 2011; Vanderleest et al., 2018). Consistent with nuclei acting as substantial mechanical barriers to internal force transmission, pulsatile dynamics are dampened specifically around the widest nuclear regions (nuclear midplanes; Fig. 5, E and F; *n* = 961) with control embryos showing a ∼24% reduction in amplitude at the midplane compared with regions basal to the nucleus. This decrease in oscillatory amplitudes is only enhanced in *kuk* embryos in which oscillatory amplitudes are dampened by 35% at the nuclear midplane (Fig. 5, E and F; *n* = 580). Thus, these results indicate the importance of nuclear deformation to intercalary behaviors and the pulsed force propagation dynamics essential for tissue extension.

### Non-dispersible nuclei lead to a deep disruption of intercalary behaviors

**Figure 6.**
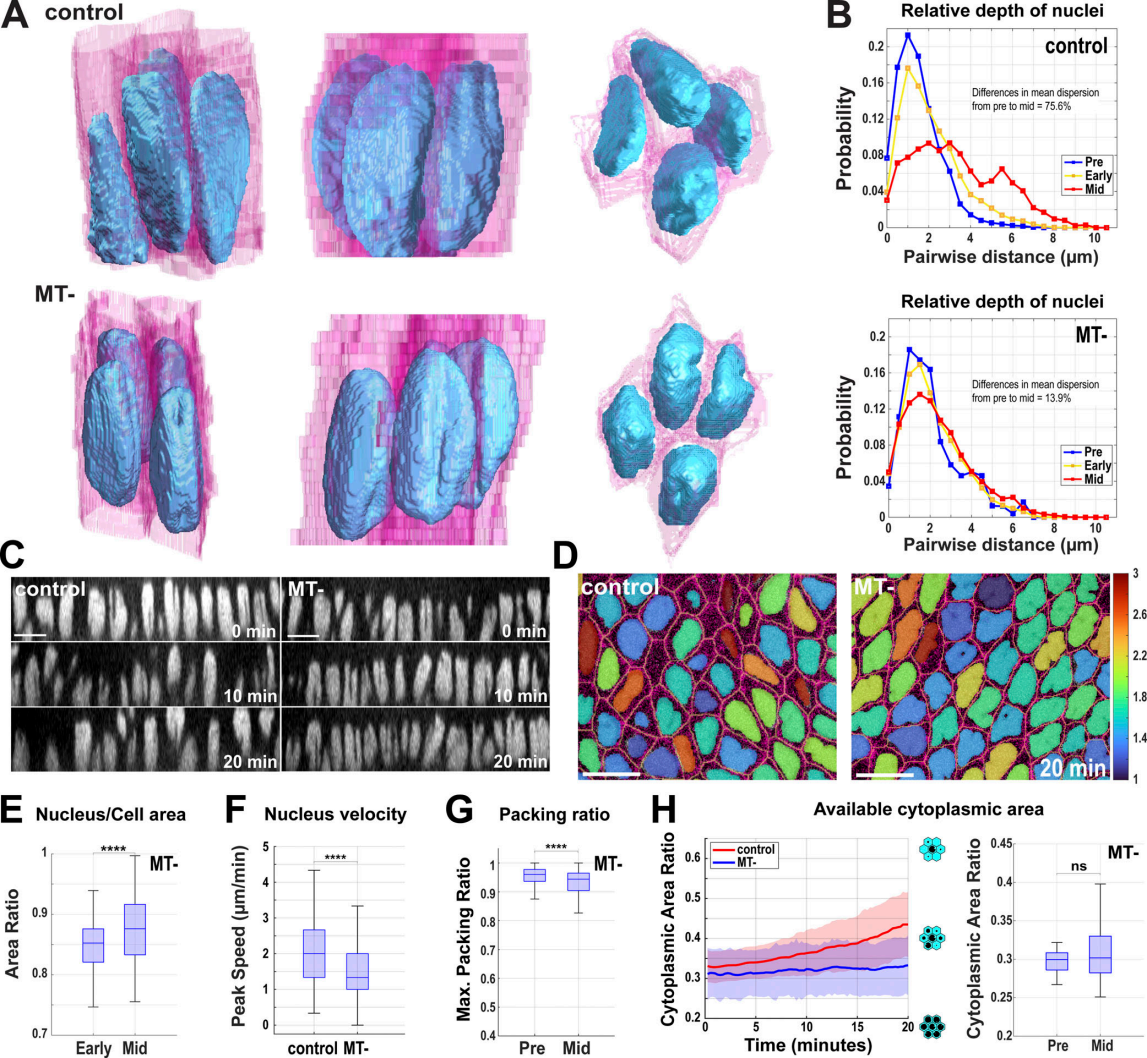
**Nuclear crowding in a common epithelial plane after MT** **disruption. (A)** 3D reconstruction of cell membranes (pink) and nuclei (blue) from live imaging data of control (vehicle-injected, top panel) or nuclear dispersion defective (colchicine-injected [MT-], bottom panel) embryos. Slanted view showing highly featured nuclear surface (left), side view (mid), and top view (right) showing nuclei flattened for optimal packing in MT- embryos (related to Video 7). **(B)** Probability of nuclear position relative to neighboring nuclei at pre- (−5 to 0 min, blue), early (0–10 min, yellow), and mid- (15–20 min, red) GBE; *n* = 10,781 pairs of nuclei from k = 3 embryos. **(C)** Orthogonal views showing decreased nuclear crowding in control (compare 0 min to 10 and 20 min) and enhanced nuclear crowding in MT- embryos (compare 0 min to 10 and 20 min). **(D)** Still image showing pavement stone-like phenotype in dispersion defective MT- embryos at late GBE (20 min) (compare to control) (related to Video 6). Nuclei are color-coded according to their major to minor axis ratio. Image slice from ∼12 µm below the apical surface. **(E)** The ratio of nucleus area to cell area at the nuclear midplane in MT- embryos compared at early (0 min) and mid- (+20 min) GBE; *n* = 290 cells and nuclei from k = 3 embryos. **(F)** Peak nuclear velocities in control and MT- embryos; *n* = 769 nuclei for control from k = 4 embryos and *n* = 325 nuclei for MT- from k = 3 embryos for each background. **(G)** Maximum packing ratio achieved by clusters of nuclei as measured at pre- (−5 min) and mid- (+20 min) GBE for MT- embryos; *n* = 420 clusters of cells from k = 3 embryos. **(H)** Average cytoplasmic area ratio available in clusters of cells at the midplane of a given central cell’s nucleus in control (red) and MT- (blue) embryos during GBE (left). Available cytoplasmic area at −5 min (pre) and +20 min (mid) after the onset of GBE (right); *n* = 370 control cell clusters from k = 4 embryos and *n* = 120 clusters for MT- from k = 3 embryos for each background. Error envelopes indicate SD. The measured *n* values are regardless of time points where not indicated. Scale bar = 10 µm for C and D. Statistical significance was calculated with Student’s *t* test in E and Mann–Whitney U-test in F–H. ns = not significant, ****P < 0.0001.

**Figure S3.**
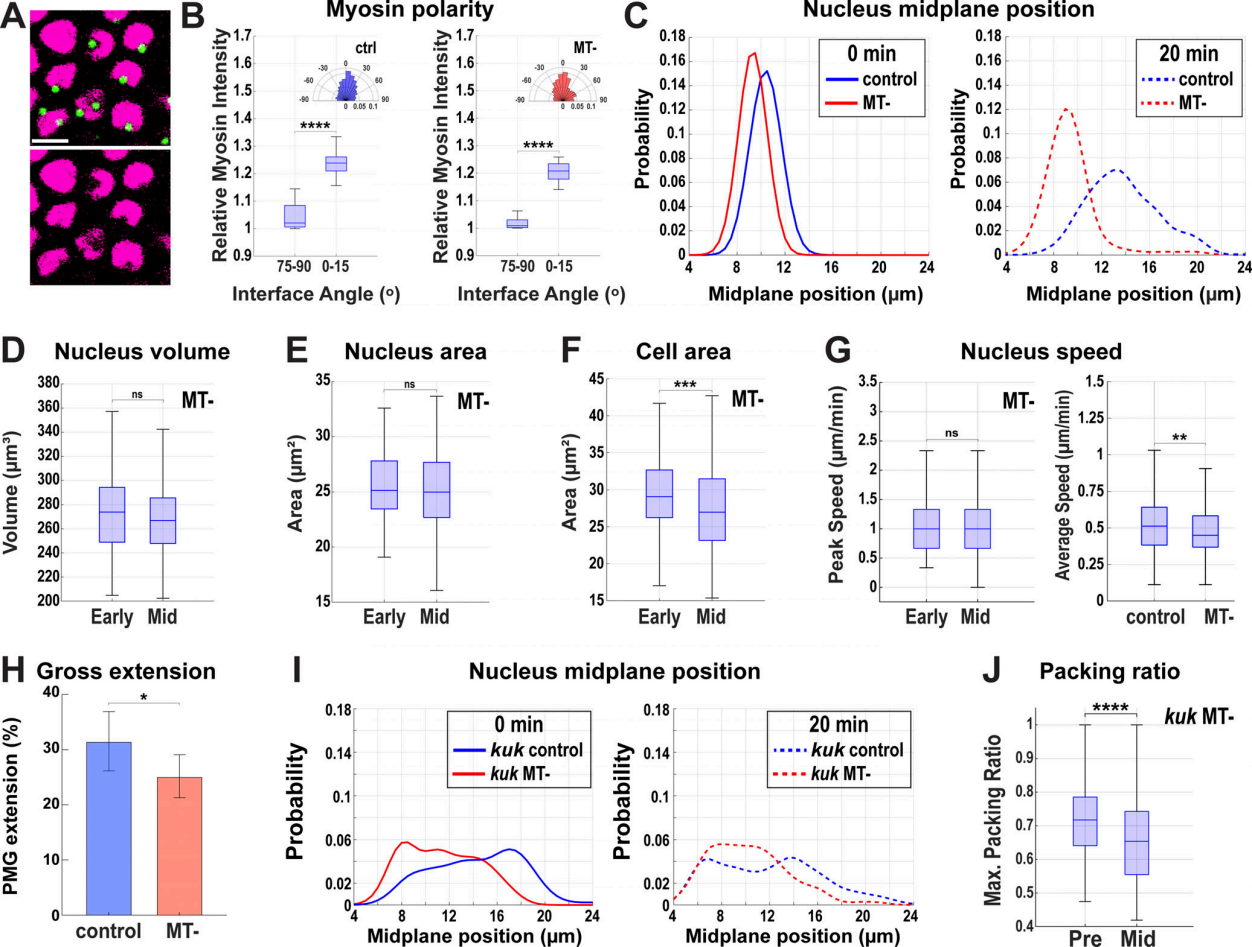
**Nuclear behaviors in MT-disrupted embryos. (A)** Still images showing centrosomes (green) and nuclei (magenta) (top) highlighting the nuclear notches (bottom) where centrosomes reside. **(B)** Comparison of relative myosin intensities at horizontal (75–90°) and vertical (0–15°) interfaces for control (vehicle-injected) and MT- (colchicine-injected) embryos; *n* = 2,289 interfaces for control and *n* = 2,342 interfaces for MT- from k = 3 embryos. Insets show rose plots indicating the directionality of the contracting interface, *n* = 2,430 interfaces for control and *n* = 1,043 interfaces for MT- from k = 3 embryos for each background. **(C)** Probability of absolute position of nuclear midplane along the apical–basal axis at 0 and 20 min of GBE for control and MT- embryos; *n* = 125 nuclei for 0 min and *n* = 132 nuclei for 20 min in control and *n* = 88 for 0 min and *n* = 98 for 20 min in MT- from k = 1 embryo for each background. **(D)** The volume of nucleus in MT- embryos compared at the early (0 min) and mid- (+20 min) GBE; *n* = 372 nuclei from k = 3 embryos. **(E)** Area of nuclei at their midplanes in MT- embryos compared at early (0 min) and mid- (+20 min) GBE. **(F)** Comparison of cell area of MT- embryos at a given z-plane at early (0 min) and mid- (+20 min) GBE. For D–F, *n* = 290 nuclei and cells from k = 3 embryos. **(G)** Maximum velocity at which nucleus move in apical–basal axis compared early (0 min) and mid- (+20 min) GBE in MT- embryos (left); *n* = 246 nuclei for early and *n* = 220 nuclei for mid-GBE from k = 3 embryos and on the right, comparing average speed of nucleus in control and MT- embryos; *n* = 554 nuclei for control and *n* = 326 nuclei for MT- from k = 3 embryos for each background. **(H)** Comparing gross extension of tissue measured by pole cell migration along the AP axis in control and MT- embryos; *n* = 6 (control) and *n* = 8 (MT-) embryos. **(I)** Probability of absolute position of nuclear midplane along the apical–basal axis at 0 and 20 min of GBE for *kuk* control (vehicle-injected *kuk*) and *kuk* MT- (colchicine-injected *kuk*) embryos; for *kuk* control, *n* = 215 nuclei for 0 min and *n* = 127 nuclei for 20 min, for *kuk* MT-, *n* = 88 for 0 min and *n* = 76 for 20 min from k = 1 embryo for each background. **(J)** Maximum packing ratio achieved by clusters of nuclei as measured at pre- (−5 min) and mid- (+20 min) GBE for *kuk* MT- embryos; *n* = 706 clusters of cells (ranging from 198 to 276 per embryo) from k = 3 embryos. The measured *n* values are regardless of timepoints where not indicated. For D–F, statistical significance was calculated using Student’s *t* test. For B and G–I, statistical significance was calculated using the Mann–Whitney U-test. ns = not significant, **P < 0.01, ***P < 0.001, ****P < 0.0001.

**Figure 7.**
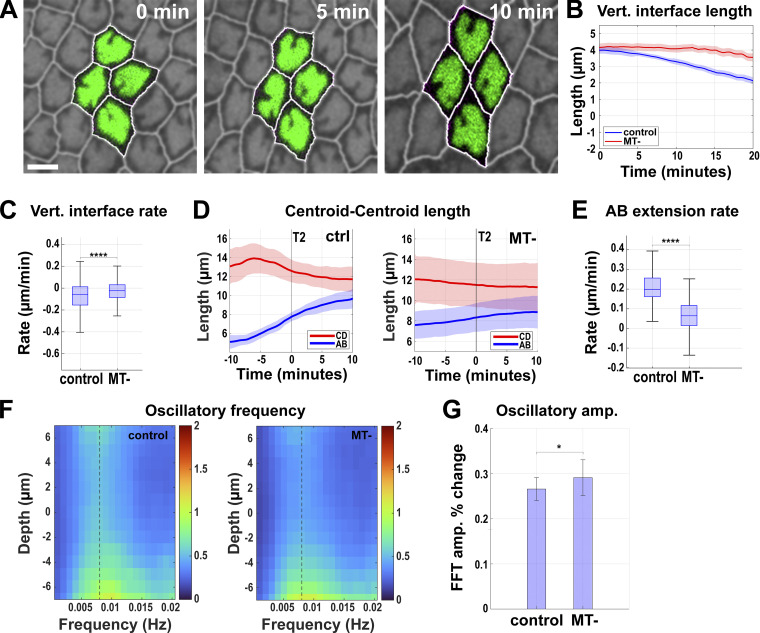
**Tissues with disrupted nuclear dispersion have deeply compromised extension and pulsatile dynamics. (A)** Still frames from a time-lapse movie of MT- embryo showing failure of nuclear dispersion indicated by nuclear crowding in the given imaging plane during a T1 cell intercalation event (related to Video 6). Image slice from ∼11 µm below the apical surface. **(B)** Comparing average length traces of vertical interfaces between control (vehicle-injected) and MT- (colchicine-injected) over the course of GBE. Error envelope indicates SEM. **(C)** Comparing the vertical interface length rate of change in control and MT- embryos. Negative values indicate contraction. For B and C, *n* = 238 interfaces for control from k = 4 embryos and *n* = 201 interfaces for MT- from k = 3 embryos. **(D)** Centroid length measurement for AB (blue) and CD (red) cells over 20 min for control and MT- embryos. Error envelopes indicate SD. **(E)** Extension rates of AB cells in control and MT- embryos. For D and E, *n* = 106 T1 transitions for control from k = 4 embryos and *n* = 87 T1 transitions for MT- from k = 3 embryos. **(F)** FFT analysis of oscillatory area changes for control and MT- embryos. The dotted line indicates the frequency at which the amplitude percent change was calculated in G. 0 μm depth indicates nuclear midplane. Positive values indicate apical and negative values indicate basal to the nuclear midplane. The color bar indicates the FFT amplitude in the frequency space. **(G)** FFT amplitude percent change from 0 μm (nuclear midplane) to −4 μm basal to the nuclear midplane at 0.008 Hz. Error bar indicates SEM. For F and G, *n* = 1,207 cells for control from k = 4 embryos and *n* = 420 cells for MT- embryos from k = 3 embryos. The measured *n* values are regardless of time points. Scale bar = 5 µm. Statistical significance was calculated using the Mann–Whitney U-test. *P < 0.05, ****P < 0.0001.

We next wanted to test the function of the nuclear dispersion pathway in driving efficient tissue elongation. As centrosomes are tightly associated with nuclei (and often are found in a depressed apical notch in the nuclear surface; Fig. 6 A and Fig. S3 A) in the germband, and as previous studies have linked nuclear movement in cells with MT function (Deshpande and Telley, 2021; Lee and Norden, 2013), we examined nuclear positioning after colchicine-induced MT disruption (MT-). Importantly, contractile amplitudes after acute colchicine injection are at, or above, the levels observed in control embryos in regions outside of where nuclei are positioned (Fig. 7, F and G), and myosin polarities and directionality of contracting interfaces are maintained in MT- embryos (Fig. S3 B). Nuclei in these embryos indeed fail to disperse significantly during GBE and they maintain their preferred initial position in a common subapical plane (Fig. 6, B and C; and Fig. S3 C; and Videos 6 and 7). Nuclear accumulation in apical planes only increases with time, leading to a “pavement stone”–like appearance in cross-section, with nuclei occupying almost all available space (89% of cross-sectional area at nuclear mid-planes 20 min into GBE; Fig. 6, D and E; and Fig. S3, C–F; and Video 6). Nuclear velocities do not change significantly at the onset of GBE, and peak and average velocities are reduced when compared with control-injected embryos (Fig. 6 F and Fig. S3 G). Consequently, the tissue experiences high nuclear density with levels surpassing 90% throughout GBE (Fig. 6 G). In control-injected embryos, the dispersion pathway decreases nuclear crowding and frees a remarkable amount of cytoplasmic area for morphogenetic movements—the average cytoplasmic area at the nuclear midplane in neighboring cells increases by almost 33% in the first 20 min of tissue extension as nuclei stagger their locations along the apical–basal axis (see schematics, Fig. 6 H). However, in non-dispersible embryos, this metric does not show any significant increase in available cytoplasmic areas (Fig. 6 H; *n* = 370 control cell clusters, *n* = 120 MT-disrupted cell clusters). In total, the absence of dispersion in these embryos, as well as the increased dispersion observed after *kuk* disruption, suggests intercalating cells use a MT-based system to distribute nuclei in 3D in response to the initiation of morphogenetic-driven cell movements.

**Video 6.** **Absence of nuclear dispersion in apical–basal dimensions after perturbation of MT function.** Nuclei display a “pavement-stone-like” phenotype and are in a common apical plane after MT disruption. Live imaging of colchicine-injected embryos expressing RFP:NLS (false-colored green) and Spider:GFP (magenta). Each frame is acquired every 15 s and the video is played at 20 frames per second. Scale bar = 10 μm.

**Video 7.** **Non-dispersible nuclei pack tightly together in a common apical plane.** 3D segmentation generated from MT- embryos (colchicine-injected) at mid-GBE expressing RFP:NLS (colored blue) and Spider:GFP (semi-transparent cell outlines, pink). The video displays two rotations of segmented cells and nuclei as shown in Fig. 6 A and demonstrates flattened nuclei due to their inability to disperse. 30 frames/second. Scale bar = 5 μm.

Given this deep disruption in nuclear dispersion, we then examined the impact of having non-dispersible nuclei on interface contraction and tissue extension. Intercalary behaviors in non-dispersible embryos were deeply affected, with vertical interface contraction rates reduced by 60% as compared with control rates (Fig. 7, A–C). Tissue extension is also greatly impacted, with the extension metric decreased by 67% (Fig. 7, D and E; P < 0.0001, *n* = 106 T1 transitions in control, *n* = 87 in MT-), which is also reflected in a significant reduction of gross movement of the germband posterior tip (Fig. S3 H). Finally, we examined contractile oscillatory dynamics necessary for changes in cell shape. Embryos with non-dispersible nuclei have a dampening of pulsed cell oscillations in regions where nuclei are widest (∼30% at the nuclear midplane), similar to control water-injected embryos (Fig. 7, F and G; P < 0.05, *n* = 1,207 cells for control and 420 cells for MT-). However, as noted above, the observed defects in interfacial remodeling are not due to an overall decrease in contractile force generation after MT disruption (Fig. 7 F). These combined data reveal the critical importance of being able to displace nuclei into different apical–basal planes for effective changes in cell dimensions and cell–cell neighbor relationships to occur.

### Disruption of deformation and dispersion pathways lead to local cell crowding and apical exclusion

**Figure 8.**
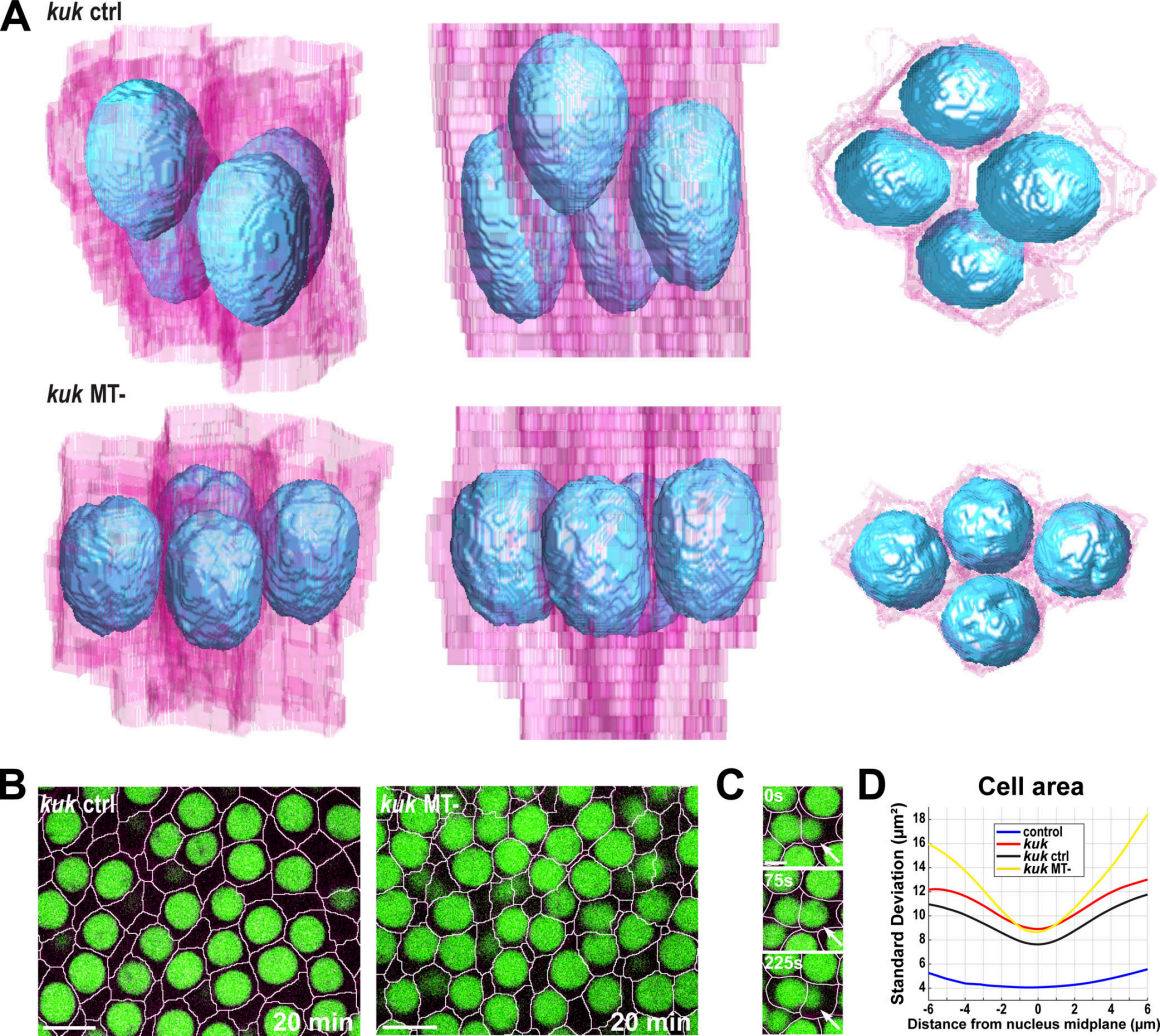
**Disruption of both the dispersion and deformation pathways causes nuclear and cell packing defects. (A)** 3D reconstruction of cell membranes (pink) and nuclei (blue) from live imaging data of non-deformable vehicle-injected (*kuk* ctrl, top panel) and non-deformable colchicine-injected *kuk* (*kuk* MT-, bottom panel) embryos (related to Video 8). **(B)** Still images showing higher nuclear crowding in apical layers in *kuk* MT- embryos as compared with *kuk* ctrl embryos at +20 min (related to Video 9). **(C)** Still images showing a rapid tug-of-war between nuclei to occupy apical space (related to Video 10). The nucleus marked by arrow initially basal to the adjacent nuclei (0 s) is pushed to the apical position (75 s) and is again forced to sink basally (225 s) due to the competition to occupy limited apical space. **(D)** SD of cell areas for given apical–basal plane in control (*luciferase* shRNA) (blue), *kuk* (red), *kuk* ctrl (black), and *kuk* MT- (yellow) embryos; *n* = 961 cells for, *n* = 580 cells for *kuk*, *n* = 1,078 cells for *kuk* ctrl, and *n* = 695 cells for *kuk* MT- from k = 3 embryos for each background. 0 μm indicates nuclear midplane, negative values indicate apical, and positive values indicate basal to the nuclear midplane. The measured *n* values are regardless of time points. For B and C, scale bar = 10 and 5 µm, respectively.

Lastly, we examined epithelial behaviors and tissue elongation when both the deformation and dispersion pathways are compromised (Fig. 8 A). These embryos revealed several interesting features, including the emergence of new crowding and extrusion phenotypes. Similar to dispersion-only defective embryos, these nuclei lack the ability to stably disperse and thus attempt to pack into a common apical plane when both nuclear-accommodation pathways are disrupted (Fig. 8 B; and Fig. S3, I and J; and Videos 8 and 9). However, the presence of non-deformable nuclei appears to progressively challenge the ability of nuclei to pack together and rapid wobbles in apical–basal positioning are observed, suggestive of internuclear tensions during nuclear crowding in apical regions (Fig. 8 C, marked by arrows; Video 10). These crowding effects also appear to impact cellular dimensions, as an increased variation in cell area occurs in these embryos as compared with controls (Fig. 8 D).

**Video 8.** **Nuclear crowding after double disruption of deformation and dispersion pathways.** 3D segmentation generated from *kuk* MT- embryos at early GBE stage expressing marker for nucleus (RFP:NLS) and plasma membrane (Spider:GFP). The video displays two rotations of segmented cells and nuclei as shown in Fig. 8 A and highlights non-deformable nuclei crowding the apical layer. 30 frames/second. Scale bar = 5 μm.

**Video 9.** **Nuclei attempt to pack tightly into apical layers after double disruption of deformation and dispersion pathways.** Live imaging of *kuk* MT- embryos expressing RFP:NLS (false colored green) and Spider: GFP (magenta). Each frame is acquired at every 15 s and video is played at 20 frames per second. Nuclei attempt to pack together in a common apical layer but lack of deformation inhibits packing and the uniformity of cell dimensions (note small cells lacking nuclei in imaged plane). Scale bar = 10 μm.

**Video 10.** **A nuclear tug-of-war to occupy the limited apical space after double disruption of deformation and dispersion pathways.** Live imaging of *kuk* MT- embryos expressing RFP:NLS (false colored green) and Spider: GFP (magenta) zoomed to show tug-of-war behaviors. Each frame is acquired at every 15 s and video is played at 20 frames per second. Scale bar = 5 μm.

**Figure 9.**
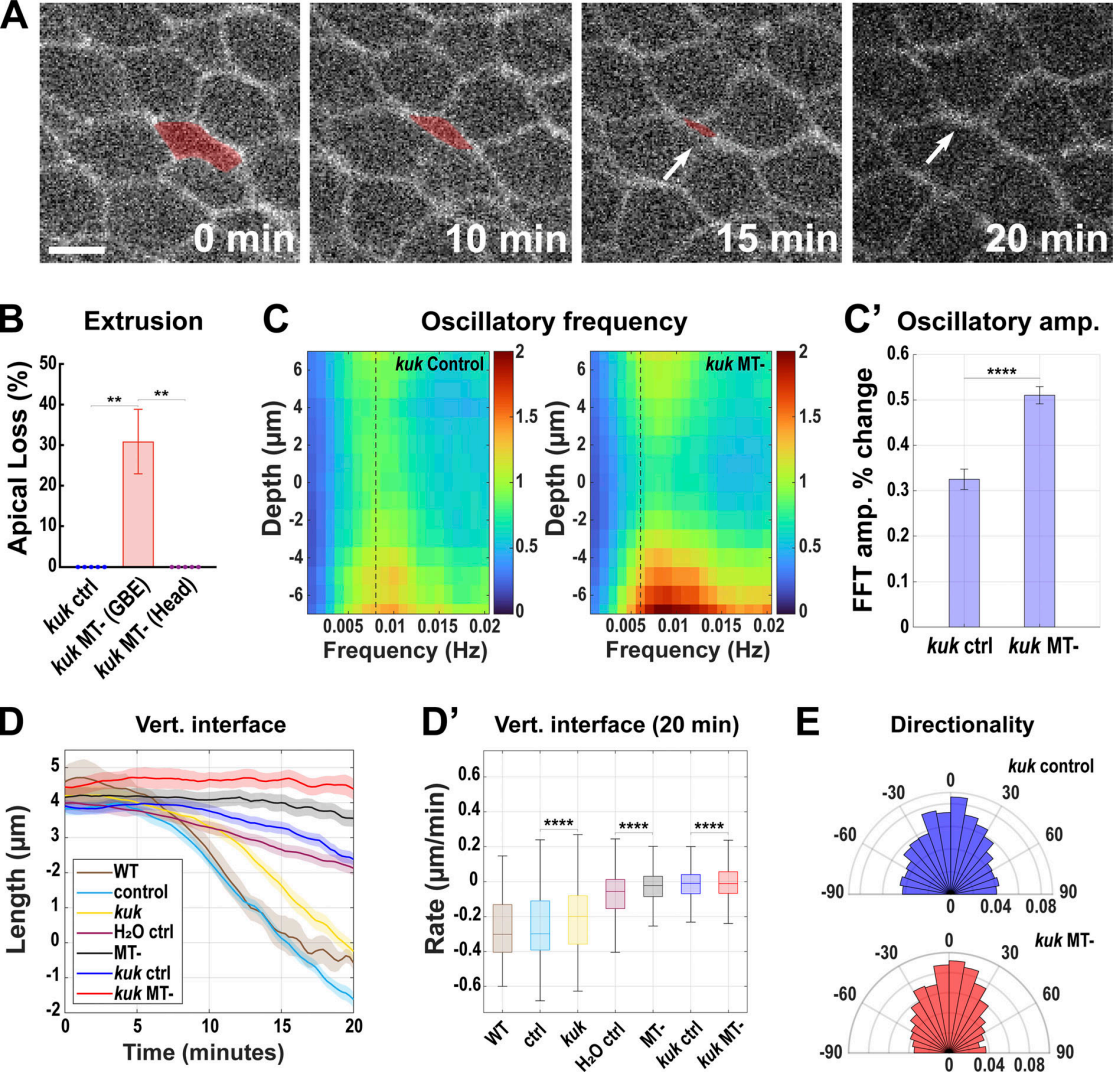
**Cells with non-deformable and non-dispersible nuclei are extruded to relieve packing. (A)** Still images showing cell extrusion-like event. The area occupied by the extruding cell (red) is gradually lost during GBE (0, 10, 15 min) before it finally disappears (arrow at 20 min). Image slice from ∼6 µm below the apical surface. Scale bar = 5 µm. **(B)** Quantitation of extrusion-like events (percent apical loss) in GBE of *kuk* MT-, *kuk* ctrl, and head (non-GBE region) of *kuk* MT- embryos; *n* = 15 measured areas from k = 3 embryos for each background. Error bar indicates SD. **(C)** FFT analysis of oscillatory area changes in *kuk* ctrl and *kuk* MT- embryos. 0 μm depth indicates the nuclear midplane, positive values indicate apical, and negative values indicate basal to the nuclear midplane. The dotted line indicates 0.008 Hz from which FFT amplitude percent change was calculated for C′. The color bar indicates the FFT amplitude in the frequency space. **(C′)** Comparison of FFT amplitude percent change between *kuk* ctrl and *kuk* MT- embryos. For C and C′, *n* = 1,078 cells for *kuk* ctrl and *n* = 695 cells for *kuk* MT- from k = 3 embryos. **(D)** Vertical interface length traces from the onset (0 min) to 20 min after GBE for wildtype (WT), *luciferase* shRNA (ctrl), *kuk*, water-injected control (H_2_O ctrl), colchicine-injected (MT-), water-injected *kuk* control (*kuk* ctrl), and colchicine-injected *kuk* (*kuk* MT-). Error envelopes indicate SEM. **(D′)** Rate of change of vertical interface lengths for all the backgrounds as in D calculated over 20 min. Color codes of boxes are the same as D. For both D and D′, *n* = 81 interfaces for WT, *n* = 168 interfaces for control (*luciferase*), *n* = 125 interfaces for *kuk*, *n* = 238 interfaces for H_2_O ctrl, *n* = 201 interfaces for MT-, *n* = 287 interfaces for *kuk* H_2_O, and *n* = 180 interfaces for *kuk* MT- from k = 4 for WT and H_2_O-injected and k = 3 for all other backgrounds. **(E)** Rose plots showing directionality of contracting interface in *kuk* control and *kuk* MT-; *n* = 1,755 interfaces for *kuk* ctrl and *n* = 1,707 for *kuk* MT- from k = 3 embryos for each background. The measured *n* values are regardless of timepoints where not indicated. Statistical significance was calculated using the Mann–Whitney U-test. **P < 0.01, ****P < 0.0001.

Additionally, as embryos continue to develop, the induced local cell crowding is often followed by a while new behavior. Embryos with both the dispersion and deformation pathways compromised display a basal cell extrusion-like behavior specific to germband cells (Fig. 9, A and B), which does not occur in any of the previously examined backgrounds. ∼30% of cells in these embryos lose contact with the apical surface and sink basally, disappearing from imaging planes (Fig. 9, A and B). These embryos still possess active contractile oscillations, but here nuclei caused a severe disruption of oscillatory propagation, with a 51% decrease in oscillation amplitudes at the nuclear midplane (compared to 33% in control embryos; Fig. 9, C and C′; P < 0.0001, *n* = 1,078 cells for *kuk* control and 695 for *kuk* MT-). The contraction dynamics of vertical interfaces were also deeply compromised in this background. While control and *kuk* vertical interfaces had negative median contraction rates (−0.30 and −0.20 μm/min, respectively, with negative numbers indicating interface shortening), when either dispersion defective (−0.02 μm/min) or both the dispersion and deformation defective (−0.007 μm/min) embryos were examined, the ability of cells to contract cell interfaces and transition into a new topological conformation was severely disrupted. Vertical, T1 interfaces are thus largely maintained in these backgrounds (Fig. 7, D and D′). Myosin polarity after *kugelkern* perturbation or colchicine injection appears to be intact, as does the directionality of interface contraction in *kuk* MT- embryos, indicating that defects in the tissue extension are the outcome of taking away both the deformation and dispersion pathways of nuclear accommodation (Fig. 9 E, Fig. S2 G, and Fig. S3 B). The above data reveal that the simultaneous disruption of dispersion and deformation pathways causes the embryonic epithelium to become increasingly unstable, which promotes extrusion-like events and deep failures in tissue remodeling and contractile behaviors.

## Discussion

**Figure S4.**
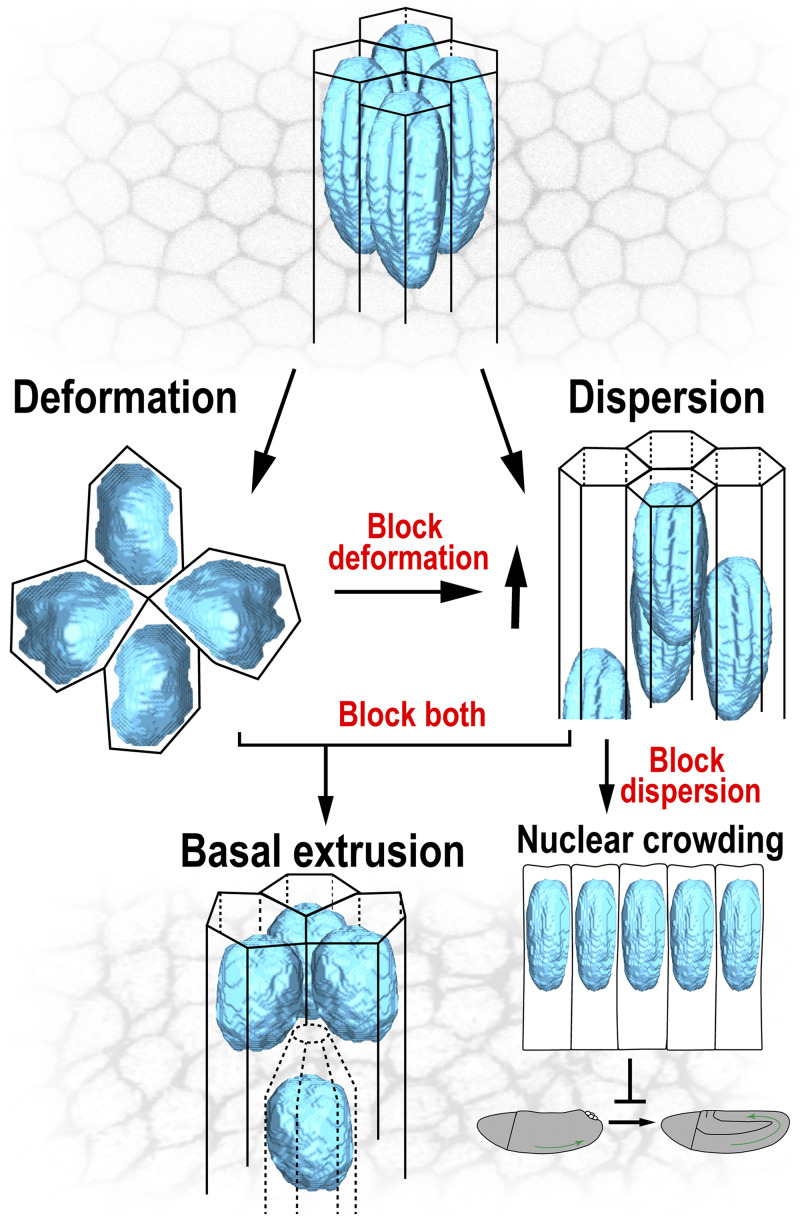
**A model of nuclear behaviors during tissue remodeling.** At the initiation of tissue extension and intercalary behaviors, nuclear volumes are accommodated through either of two pathways—deformation or dispersion. Dispersion pathways may be more essential to intercalation as planar nuclear tensions appear to be particularly reduced by dispersion as compared to deformation. Perturbing nuclear deformation causes enhanced levels of nuclear dispersion, and blocking dispersion creates a strong block in tissue extension dynamics as nuclear crowding increases. A new “extrusion-like” phenotype arises when both pathways are made unavailable for the nuclei.

In total, these results demonstrate how nuclear plasticity, in both shape and location, is essential for epithelial morphogenesis. Failures in either of the pathways that permit the accommodation of nuclear volumes lead to defects in the ability of cells to undergo neighbor exchange movements necessary for cell intercalation (model in Fig. S4). The inability to properly position or deform nuclei also affects the regularity of epithelial shapes, with cells forced to warp cellular dimensions to adapt to nuclear bulk, causing large variations in cell organization across the epithelium. This reveals the degree to which the nucleus represents an internal physical impediment even to processes that function at cortical and cell surface regions. Indeed, the oscillatory contractions that help power cell shaping events are specifically dampened where nuclei most closely approach the cell cortex.

Our results demonstrate that cells undergoing tissue extension in the early embryo use two orthogonal processes to relieve stresses created by nuclear crowding: nuclear deformation in the plane of the epithelium (x-y imaging plane) and nuclear dispersion in the apical–basal axis (z-imaging plane). Both processes are ways of filling the available cellular space more efficiently and should thus reduce the amount of mechanical interaction between tightly packed neighboring nuclei. During intercalation, GBE cells experience shear forces (and shear motion) in the x–y plane of the epithelium, but not in the apical-basal z-direction, as cells undergo planar polarized neighbor exchange (Kale et al., 2018). It is tempting to speculate that this asymmetry may underlie why disruption of the two accommodation mechanisms differentially affects intercalation and tissue extension and could be why the dispersion pathway is the more potent approach to relieve internuclear stresses in a planar intercalating epithelium. Our work could also be viewed from the perspective of a tissue fluidization process (in the framework of a jamming/unjamming transition), where the nuclear dispersion and deformation processes help to “unjam” the early epithelium. This unjamming of nuclei permits a flow state for the elongation of the tissue, and our results indicate the unique contributions of nuclear deformation and positioning pathways to this state.

We would also note that work across a variety of systems has indicated that MT-based processes are often involved in the regulation of nuclear positioning. These processes often use linker proteins (such as SUN/KASH proteins) embedded in the nuclear membrane that connects the inner-nuclear lamins to the MT cytoskeleton for nuclear transport. During vertebrate neurogenesis, interkinetic migration of radial glial progenitor cells has been shown to be driven by MT-based transport with velocities of around 0.1–0.2 µm/min (Tsai et al., 2010), while other neuronal systems show velocities that range from 0.1 to 1.5 µm/min (Solecki et al., 2004; Tsai et al., 2007). Our average velocities (0.47 μm/min) fit into a similar range, although we observed peak velocities as high as 2.0 µm/min. It is also intriguing to note that a similar challenge to cell packing in cellular regions that possess nuclei has been observed in pseudostratified epithelia from mouse lung explants undergoing interkinetic nuclear migration (Gómez et al., 2021). Interestingly, our initial genetic analyses failed to reveal a function for SUN/KASH linker proteins in dispersion in the germband at these stages, although, for various technical reasons, the function of these proteins at these stages cannot be ruled out. However, our results suggest a similar MT dependence of dispersion to these classic systems of nuclear positioning. MTs could also contribute to intercalary dynamics in ways other than transport—for example, by promoting nuclear distancing from the cell periphery for efficient cytoplasmic flow or through mechanical connections to contractile cytoskeletal elements. Going forward, it will be important to explore the molecular regulators of dispersion, as well as whether there is a coordinated interplay between MT positioning and the cortical actomyosin forces that are present in the early epithelium. Indeed, in several types of migratory cell systems, actomyosin linkages with nuclei assist in the positioning and displacement of nuclei (Leung et al., 2011; Meyer et al., 2011; Norden et al., 2009; Rujano et al., 2013). Additionally, the greater dispersion of nuclei in the *kuk *non-deformable background is suggestive of a potential mechanosensitive element that may sense nuclear crowding.

## Materials and methods

### Live imaging and injection

Embryos were collected in apple juice agar plates, dechorionated in 50% bleach solution for 2 min, washed, then transferred to an air-permeable membrane, and covered with Halocarbon 27 oil. All time-lapse imaging was performed at 25°C on a CSU10b Yokogawa spinning disk confocal from Zeiss/Solamere Technologies Group with a 63× 1.4 NA objective captured with a Hamamatsu ORCA EMCCD or Prime 95 sCMOS camera using Micro-Manager software. For each movie, 20 z-layers at 1 μm distance were captured with a time resolution of 15 s. For absolute nuclear midplane determination, movies were captured for 30 z-layers at 1 μm distance with a time resolution of 30 s.

For small-molecule injections, after embryos were dechorionated as described above, embryos were then placed on apple juice agar and dehydrated for 12 min, covered with Halocarbon 700 oil, and injected with colchicine (Cat #C3915; Sigma-Aldrich, 1 mg/ml in H_2_O) and imaged in the same settings described above. All actomyosin forces inhibition experiment was carried out by injecting Y27632 (Cat #281642; Santa Cruz, 25 mM in H_2_O) in embryos, and time-lapse imaging was performed where 20 z-layers at 1 μm distance were captured with a time resolution of 20 s. Vehicle-injected (water) embryos were used as control. Embryos were imaged within 10 min of injection. Colchicine injected embryos showed apical accumulation of nuclei and disrupted germband extension metrics.

### qPCR for knockdown analysis

Embryos from wildtype and *kuk* flies were collected in an apple juice agar plate containing yeast for 3 h and then aged for 2 more hours. The embryos were dechorionated in 50% bleach solution for 2 min, washed, and transferred to microcentrifuge tubes. RNA extraction was carried out in biological triplicates using Zymo Research Quick-RNA Microprep Kit (Cat #11-327M; Genesee Scientific) and stored at −80°C until further use. Thus extracted RNA was reverse transcribed to obtain cDNA with the QIAGEN QuantiTect Reverse Transcription Kit (205311; QIAGEN), which was used as the template for qPCR reactions. Two primers, each targeting the CDS region and UTR region of kuk gene, were custom-designed and the oligomers were obtained from Thermo Fisher Scientific. qPCR experiments were performed using QIAGEN QuantiTect SYBR Green PCR Kit (204143; QIAGEN) and QIAGEN Primer Assays (249900; QIAGEN) for sqh (positive control) and Rh3 (negative control). Bio-Rad iQ5 Multicolor Real-Time PCR Detection System was used for the qPCR and the data analysis was done using double delta Ct method to obtain the fold expression.

### Cell segmentation

Image and data analysis were performed in MATLAB. Cells were segmented first in a single z-layer using a seeded watershed algorithm, then propagated into the remaining z-layers and tracked in time (Jewett et al., 2017; Miao et al., 2019; Vanderleest et al., 2018, 2022). For each z-layer, the skeletonized representation of the tissue directly yields cell areas, perimeters, and centroid positions, as well as vertex positions and interface lengths, which are stored along with cell–cell and vertex–vertex connectivity matrices. Interface lengths are defined as the Euclidean distances between corresponding vertices.

### Nucleus segmentation

Nuclei were segmented individually within the 3D bounding box defined by each segmented cell, first by using a simple intensity threshold to establish the rough outline of the nucleus, then adding surface features by finding connected voxels of intensity close to the median intensity of the rough nuclear segmentation. Nucleus tracking labels were inherited from their associated cells. Volumes were calculated by summing the number of voxels with the same tracking label, multiplied by the voxel volume. Nucleus lengths were determined by finding the distance along the z-axis between the first and last z-layers containing any segmented voxels. Cell and nucleus areas were measured as the sum of segmented cell or nucleus pixels, respectively, multiplied by the pixel area.

### Cytoplasmic area in neighboring cells

The cytoplasmic area in a cell at a particular z-layer was calculated by subtracting the segmented nucleus area from the cell area at that z-layer. We used the average cytoplasmic area in a cluster of cells as an inverse measure of nucleus packing. To do so, for a central cell and its immediate neighbors, we found the average cytoplasmic area in the neighbor cells at the midplane of the central cell’s nucleus and then divided that by the average area of the cells in the cluster to get a measure of the typical proportion of free space visible to the central nucleus. Lower ratios indicate greater nuclear packing and vice versa.

#### 2D aspect ratio

The aspect ratio of cells and nuclei was found using the MATLAB *regionprops* function to obtain the major and minor axis lengths of each cell and nucleus at a given z-layer. The aspect ratio is defined as the major axis length divided by the minor axis length.

#### Shape factor

For 2D shapes, shape factor refers to the circularity of an object, defined as $SF=\frac{P^{2}}{4\pi A}$, where *P* and *A* are the perimeter and area of the object, respectively, such that *SF* = 1 for a perfect circle, and increases for non-circular shapes. The perimeter and area for each cell/nucleus were found at the nucleus midplane using the *regionprops* function in MATLAB.

### Nuclear dispersion

#### Midplane identification

For the purpose of tracking nuclear dispersion along the apical–basal axis of cells, we required a consistent reference point to determine the nucleus position. Because the distribution of nuclear volume can significantly shift up or down during GBE (making nuclei that are top- or bottom-heavy), the widest point of the nucleus is not an ideal indicator of position. Instead, we defined a “midplane” for each nucleus. The position of the nucleus midplane was determined by first finding the plane with the maximum area for a given nucleus and then finding the planes on either side with the area closest to 50% of the maximum. The midplane is defined as the midpoint between those two positions. Absolute nucleus depth was measured before the onset of germband extension in movies with imaging planes starting above the apical surface of the embryo and defined as the distance along the z-axis between the nucleus midplane and the first z-layer where apical cell caps are visible. Because the apical surface of the embryo buckles and forms local depressions over time as shaping forces are exerted, in most cases, nuclear dispersion is characterized by the relative distance between the midplanes of neighboring nuclei.

#### Nuclear midplane positions

The majority of data reported in this manuscript consists of an imaging volume that spans from apical regions to ∼20 μm in depth. When absolute nuclear midplane positions were measured, the analyses were performed on a larger volume that included the embryo’s apical surface and the resultant slower temporal resolution. These data have ∼30 μm of captured depth with a 30-s time resolution. We define absolute nuclear positions as the distance from the apical surface of the cell to the nuclear midplane, calculated from these large-volume datasets.

#### Packing of nucleus midplanes

We defined the degree to which nuclei in neighboring cells were packed into a common plane as $packing\left(z\right)=\frac{\sum A\left(z\right)}{\sum \max\;A}$, where *A*(*z*) is the cross-sectional area of a nucleus at a given z-plane and *maxA* is the largest cross-sectional area of that nucleus in all z-planes, with the summation over all participating nuclei, such that when the maximum areas of a cluster of nuclei all align in the same plane, *packing*(*z*) = 1.

#### Nucleus velocity

Movies were qualitatively assessed to ensure minimal Z-shifting throughout the analysis window (up to 20 min after the onset of GBE). We tracked the midplane position of each nucleus relative to the apical-most z-layer cell surface over time and calculated the apical–basal velocities calculated over a 1-min time window for wildtype embryos. Drug- and vehicle-injected embryos tend to develop more slowly and therefore have the most meaningful velocity changes over slightly longer time intervals compared with non-injected embryos. Because of this, velocities for injected embryos were calculated over a 2-min time window. In the included peak and average velocity boxplots, each tracked nucleus contributes a single data point, representing its max/mean velocity respectively.

#### MSD of relative position

We used MSD to discern active transport during nuclear positioning. MSD is the customary method to characterize a trajectory as active, diffusive, or constrained based on whether the MSD curves upward, is linear, or curves downward, respectively. The MSD for distance is defined as $MSD\left(\tau \right)=\frac{1}{t-\tau }\overset{t-\tau }{\underset{k=1}{\sum }}{\left[d\left(k+\tau \right)-d\left(k\right)\right]}^{2}$, where *t* is the length of the distance trajectory, *τ* is the time lag between frames, and *d* is the distance between midplanes for a pair of nuclei.

### Nuclear geometry

#### Nucleus sphericity

As epithelial nuclei are primarily elongated along the apical–basal axis of the cell, we used a unitless measure of nucleus sphericity based on length and area at the midplane, defined as $sphericity=\frac{L\sqrt{\pi }}{2\sqrt{A}}$, where *L* is the nucleus length along the z-axis and *A* is the area of the nucleus at its midplane. The $\frac{\sqrt{\pi }}{2}$ term scales the value such that *sphericity* = 1 for a perfect sphere.

### Extension directionality

The directionality of tissue extension was quantified by isolating contractile interfaces (those with a length change rate less than or equal to −0.5 µm/min) and plotting the angle distributions of said interfaces in a polar coordinate system. Interfaces with angles of 0° indicate vertical interfaces (aligned with the DV axis) that canonically contract/shrink in length, while interface angles of ±90° represent horizontal interfaces (aligned with the AP axis) that canonically grow in length.

### Contraction and extension rates

The average rate of change for T1–T3 interface length and AB cell centroid distance was calculated by taking the difference between the mean values of interface length/centroid distance at 10 min before and after the T2 time point and then dividing by 20 min. Length rates of change for vertical interfaces (i.e., those not limited to T1 transitions) were similarly calculated by taking the difference between interface lengths at *t* = 0 and *t* = 20 min after the onset of GBE and then dividing by 20 min. For the length measurements, positive lengths indicate T1 interfaces while negative lengths are horizontally extending T3 interfaces. For the vertical interface length rate of change measurements, negative values indicate the contraction of vertical interfaces.

### Fast fourier transform (FFT)

FFT-based analysis was performed on a cell area aligned to the midplane of the associated nucleus. The area at each z-layer was measured independently, processed for signal detrending, and a 1D FFT was performed on all signals that had a duration of at least 3 min. The oscillation heatmaps shown throughout the manuscript are color-coded by the FFT amplitude in the frequency space. In general, the higher the amplitude of a signal in the frequency domain is, the stronger that certain frequency is in the time domain. FFT amplitude peaks describe the strongest and most coherent frequency contributions of a signal.0 μm in depth indicates the nuclear midplane, positive values indicate apical, and negative values indicate basal to the nuclear midplane.

### Measurement of extrusion-like events

Several regions (five regions per embryo) of the identical area in the ventrolateral germband (100 × 100 pixels; 1 pixel = 0.16 µm) or the embryo head (100 × 100 pixels; 1 pixel = 0.28 µm) were drawn and observed in a representative apical layer (z-layer = 6) using ImageJ software. The number of cells in each region was counted at different time points (0, 20, 40, and 55 min for GBE and 0 and 20 min for head prior to the onset of mitotic domains) for each movie and documented in Microsoft Excel. The number of extrusion-like events appearing in that region was counted along with the cell number, and the percent extrusion was calculated by dividing the number of extrusion events by the number of cells.

### Myosin intensities

#### Interfacial myosin intensity ratio

Planar polarity of myosin intensities along interface was automatically measured in MATLAB along regions of interest (ROIs) produced from the cell segmentation, with the skeletonized interface between each pair of cells dilated to define a roughly eight pixel-wide ROI around each interface. The values in the myosin channel of pixels within the ROI were averaged to define the mean myosin intensity of an interface at each depth, and then the maximum value over all depths was used as the final intensity value for that interface. The maximum intensity values were normalized to the mean myosin intensity in each frame. Quantification was performed by binning the intensity values of interfaces from every embryo within a set range of angles, where θ = 0° corresponds to a DV-axis aligned “vertical” interface, and θ = ±90° corresponds to an AP-axis aligned “horizontal” interface.

#### Medial myosin intensities

Medial myosin intensities were similarly measured in MATLAB. ROIs were defined as the centralmost region of each cell, isolated by excluding a dilated region around segmented cell contact. Intensities were extracted from a max projection of the myosin channel over the four apical-most Z-layers, or 2 μm of depth. Background fluorescence was subtracted from the resultant per-cell intensities. To accommodate for significant cell size differences between backgrounds, each cell’s medial myosin intensity was divided by its area to reflect a per-μm intensity value.

### Image editing and figure preparation

Spinning disk images were edited with Fiji, ImageJ, or Adobe Photoshop, and the images were leveled identically between samples for optimal appearances. All embryos were oriented with an anterior left, posterior right, dorsal up, and ventral down in the figure. The graph in Figs. S3 H and 9 B was generated in GraphPad Prism. Figures were prepared and labeled in Adobe Illustrator.

### Fly stocks and genetics

UAS-kuk TRiP Valium 22, 41872 and UAS-pLuc Valium 10, 35788 (Bloomington Drosophila Stock Center [BDSC]); Spider:GFP (A. Debec, Paris Diderot University, Paris, France); ubi:RFP:NLS (BDSC # 30555; 34500); matαTub-Gal4VP16 67C;15 (D. St. Johnson, Gurdon Institute, Cambridge, UK). UAS transgenic flies were crossed to matαTub-Gal4VP16 67C;15 maternal driver females and second-generation embryos were analyzed. We used FlyBase (FB2020 to FB2023) for information on genes, phenotypes, function, stocks, gene expression, and more.

### Online supplemental material

Fig S1 shows the quantitation of cell and nuclear behaviors in wildtype and their comparison in control and myosin-disrupted epithelia. Fig S2 shows the features of cells and nuclei compared in control and kuk perturbed embryos. Fig S3 shows the comparison of nuclear behaviors in control and MT perturbed embryos. Video 1 highlights the 3D structure of nuclei in the epithelial cells at early GBE. Video 2 reveals one of the two mechanisms of nuclear accommodation during GBE, i.e., nuclear deformation during dynamic cell shape changes. Video 3 unveils the second mechanism of nuclear accommodation during GBE, i.e., the dispersion of nuclei to different apical–basal planes. Video 4 shows how the nuclei in *kuk* embryos resist deformation compared to the nuclei in control embryos. Video 5 displays 3D structure of nuclei in *kuk* embryos, highlighting their smooth surfaces, shorter height, and rounder shapes. Video 6 shows the failure of nuclei to disperse in an apical–basal plane during GBE when MT function is chemically inhibited. Video 7 illustrates the 3D structure of nuclei in epithelial cells at mid-GBE when MT function is perturbed. Video 8 reveals the distortion of cell shape in 3D due to the presence of non-deforming non-dispersing nuclei in the common apical plane. Video 9 shows the attempt of nuclei to pack tightly in the apical region after double disruption of deformation and dispersion pathways. Video 10 exemplifies the nuclear tug-of-war to occupy the limitedly available apical space after double perturbation of deformation and dispersion pathways.

## Data Availability

All measurements were quantified from a minimum of three embryos and represent at least two individual trials. All statistical analysis and graph generation were done using GraphPad prism 9.0.0. Student’s *t* tests were two-sided and a Kolmogorov–Smirnov test was used for normality. All box and whisker plots represent 25th quartile (bottom of the box), median (mid of the box), and 75% quartile (top of the box), and the whiskers represent the minimum (below the box) and the maximum (above the box) values. All MATLAB coding and algorithms, as well as primary data, are freely available from the corresponding author upon reasonable request.
